# Solving optimization problems simultaneously: the variants of the traveling salesman problem with time windows using multifactorial evolutionary algorithm

**DOI:** 10.7717/peerj-cs.1192

**Published:** 2023-01-10

**Authors:** Ha-Bang Ban, Dang-Hai Pham

**Affiliations:** Computer Science Department, School of Information and Communication Technology, Hanoi University of Science and Technology, Hanoi, Vietnam

**Keywords:** MFEA, TSPTW, TRPTW, Metaheuristic, Algorithm, Combinatorial optimization

## Abstract

We studied two problems called the Traveling Repairman Problem (TRPTW) and Traveling Salesman Problem (TSPTW) with time windows. The TRPTW wants to minimize the sum of travel durations between a depot and customer locations, while the TSPTW aims to minimize the total time to visit all customers. In these two problems, the deliveries are made during a specific time window given by the customers. The difference between the TRPTW and TSPTW is that the TRPTW takes a customer-oriented view, whereas the TSPTW is server-oriented. Existing algorithms have been developed for solving two problems independently in the literature. However, the literature does not have an algorithm that simultaneously solves two problems. Multifactorial Evolutionary Algorithm (MFEA) is a variant of the Evolutionary Algorithm (EA), aiming to solve multiple factorial tasks simultaneously. The main advantage of the approach is to allow transferrable knowledge between tasks. Therefore, it can improve the solution quality for multitasks. This article presents an efficient algorithm that combines the MFEA framework and Randomized Variable Neighborhood Search (RVNS) to solve two problems simultaneously. The proposed algorithm has transferrable knowledge between tasks from the MFEA and the ability to exploit good solution space from RVNS. The proposed algorithm is compared directly to the state-of-the-art MFEA on numerous datasets. Experimental results show the proposed algorithm outperforms the state-of-the-art MFEA in many cases. In addition, it finds several new best-known solutions.

## Introduction

### The TSPTW and TRPTW literature

The Traveling Salesman Problem with Time Windows (TSPTW) (Gendreau et al., 1998; Silva & Urrutia, 2010; Ohlmann & Thomas, 2007), and Traveling Repairman Problem with Time Windows (TRPTW) (Ban & Nghia, 2017; Ban, 2021; Heilporna, Cordeaua & Laporte, 2010) are combinatorial optimization problems that have many practical situations. The TRPTW wants to minimize the sum of travel durations between a depot and customer locations, while the TSPTW aims to minimize the total time to visit all customers. In the two problems, the deliveries are made during a specific time window given by the customers. Due to time window constraints, the TSPTW and TRPTW are much harder than the traditional Traveling Salesman Problem (TSP) and Traveling Repairman Problem (TRP).

The Travelling Salesman Problem with Time Windows (TSPTW) is a popular NP-hard combinatorial optimization problem studied much in the literature (Dumas, Desrosiers & Gélinas, 1995). The algorithms include exact and metaheuristic approaches. Langevin et al. (1993) introduced a two-commodity flow formulation to solve the problem. Dumas, Desrosiers & Gélinas (1995) then used a dynamic programming approach. Similarly, Gendreau et al. (1998) brought constraint programming and optimization techniques together. Most recently, Dash et al. (2012) propose a method using an IP model based on the discretization of time. The results are extremely good: several benchmark instances are solved first. Gendreau et al. (1998) then proposed an insertion heuristic that generated the solution in the first step and improved it in a post-phase using removal and reinsertion of vertices. Ohlmann & Thomas (2007) developed simulated annealing relaxing the time windows constraints by integrating a variable penalty method within a stochastic search procedure. In this work, they developed a two-phase heuristic. In the first phase, a feasible solution was created by using a Variable Neighborhood Search (VNS) (Mladenovic & Hansen, 1997). In the post phase, this solution was improved by using a General VNS. Generally speaking, the results from this approach are very promising.

In the Travelling Repairman Problem with Time Windows (TRPTW), there is an exact algorithm and three metaheuristics algorithms in the literature: (1) Tsitsiklis (1992) proposed a polynomial algorithm when several customers are bounded; (2) Heilporna, Cordeaua & Laporte (2010) then developed an exact algorithm and heuristic algorithm to solve the problem; (3) Ban & Nghia (2017) and Ban (2021) proposed two metaheuristic algorithms based on Variable Neighborhood Search (VNS) scheme. Their experimental results showed the efficiency of the metaheuristic approach.

These above algorithms are the state-of-the-art metaheuristics in current. However, they are designed to solve each problem independently. That means they cannot solve both two problems well simultaneously. In Salehipour et al. (2011) also showed that a good algorithm for this problem might not be good for the other problem. Therefore, developing an algorithm solving both problems well simultaneously is our aim in this work.

### The MFEA literature

To date, the MFEA framework (Dash et al., 2012; Gupta, Ong & Feng, 2016; Xu et al., 2021) has been introduced in the literature. Using a unified search space for multiple tasks can exploit good transferrable knowledge between optimization tasks. In addition, although solving many tasks simultaneously, the flow of the MFEA framework is sequential. Therefore, the MFEA is suitable in a system with limited computation.

Several close variants of the MFEA (Ban & Pham, 2022; Osaba et al., 2020; Yuan et al., 2016) are developed to solve permutation-based optimization problems such as the TSP and TRP. Therefore, they are direct references to our research. Yuan et al. (2016) firstly developed evolutionary multitasking in permutation-based optimization problems. They tested it on several popular combinatorial problems. The experiment results indicated the good scalability of evolutionary multitasking in many-task environments. Osaba et al. (2020) then proposed a dMFEA-II framework to exploit the complementarities among several tasks, often achieved *via* genetic information transfer. Their dMFEA-II controls the knowledge transfer by adjusting the crossover probability value. The technique allows good knowledge to transfer between tasks. Although the results are promising, the drawback of the above two algorithms (Osaba et al., 2020; Yuan et al., 2016) is that there is a lack of a mechanism to exploit the good solution space explored by MFEA. Therefore, these algorithms cannot effectively balance exploration and exploitation (we visualize their issue in “Comparison with the previous MFEA algorithms” in more detail). Recently, Ban & Pham (2022) have successfully applied the MFEA with RVNS to solve two problems such as TSP and TRP. Their algorithms maintain the exploration and exploitation better than the others in Osaba et al. (2020) and Yuan et al. (2016). Its performance encourages us to use this combination to solve the TSPTW and TRPTW. This article considers these works as a baseline for our research.

### Our contributions

This article introduces the first algorithm combining the MFEA framework and RVNS to solve two tasks simultaneously. The combination is to have positive transferrable knowledge between tasks from the MFEA and the ability to exploit good solution spaces from RVNS. The major contributions of this work are as follows:

- We propose a new selection operator that balances skill-factor and population diversity. The skill-factor effectively transfers elite genes between tasks, while diversity in the population is important when it meets a bottleneck against the information transfer.
- Multiple crossover schemes are applied in the proposed MFEA. They help the algorithm have good enough diversity. In addition, two types of crossover (intra- and inter-) are used. It opens up the chance for knowledge transfer through crossover-based exchange between tasks.
- The combination between the MFEA and the RVNS is to have good transferrable knowledge between tasks from the MFEA and the ability to exploit good solution spaces from the RVNS. However, focusing only on reducing cost function maybe lead the search to infeasible solution spaces like the algorithms (Ban & Pham, 2022; Osaba et al., 2020; Yuan et al., 2016). Therefore, the repair method is incorporated into the proposed algorithm to balance finding feasible solution spaces and reducing cost function.
- Numerical experiments show that the proposed algorithm reaches nearly optimal solutions simultaneously in a short time for two problems. Moreover, it obtains better solutions than the previous MFEA algorithms in many cases.

The rest of this article is organized as follows. Sections 1 and 2 present the literature and preliminary, respectively. Section 3 describes the proposed algorithm. Computational evaluations are reported in Section 4. Section 5 presents the conclusions and future work.

## The formulation and methodolgy

### The formulation

We consider an example that describes the difference between two problems in a specific instance. If we use the optimal solution of n40w160.002 instance for the TSPTW (https://lopez-ibanez.eu/tsptw-instances), the objective function cost (using the function cost of the TRPTW) of this solution for the TRPTW is 7,519, while the known-best cost for this instance for the TRPTW is 6,351 (the known-best cost is found by our algorithm). Thus, the difference between the two objective function costs is 15.5%. It implies that a good metaheuristic algorithm for the TSPTW does not produce a good solution for the TRPTW and vice versa. The above algorithms are the best algorithms for two problems. However, they only solve each problem independently but cannot simultaneously produce good solutions for two problems.

We have a complete graph ${K_n} = (V,E)$, where $V = {v_1},{v_2},...,{v_n}$ is a set of vertices showing the starting vertex and customer locations, and *E* the set of edges connecting the customer locations. Suppose that, in a tour $T = ({v_1} = s,{v_2}...,{v_n})$, each edge $({v_i},{v_j}) \in E$ connecting the two vertices ${v_i}$ and ${v_j}$ there exists a cost $c({v_i},{v_j})$. This cost represents the travel time between vertex ${v_i}$ and ${v_j}$. Each vertex ${v_i} \in V$ has a time window $[{e_i},{l_i}]$ indicating when starting service time at vertex ${v_i}$. This implies that a vertex ${v_i}$ may be reached before the start of ${e_i}$, but the service cannot start until ${e_i}$ and no later than ${l_i}$ of its time window. Moreover, to serve each customer, the salesman spends an amount of time. Let $D({v_i}),S({v_i})$ be the time at which service begins and the service time at vertex ${v_i}$. It is calculated as follows: $D({v_i}) = \max \{ A({v_i}),{e_i}\}$, where $A({v_k}) = D({v_{i - 1}}) + S({v_{i - 1}}) + c({v_{i - 1}},{v_i})$ is the arrival time at vertex ${v_i}$ in the tour. A tour is feasible if and only if $A({v_i}) \le {l_i}$ for all vertices. The objective functions of the two problems are defined as follows:

- In the TSPTW, the salesman must return to $s$. Therefore, the cost of the tour *T* is defined as: $\sum\nolimits_{i = 1}^n {c({v_i},{v_{i + 1}})}$. Note that: ${v_{n + 1}} \equiv s$
- In the TRPTW, we also define the travel duration of vertex ${v_i}$ as the difference between the beginning of service at vertex ${v_i}$ and the beginning of service at *s*: ${t_i} = D({v_i}) - D(s)$. The cost of the tour *T* is defined: $\sum\nolimits_{i = 2}^n {{t_i}.}$

Two problems consist of determining a tour, starting at the starting vertex ${v_1}$, minimizing the cost of the tour overall vertices while respecting time windows. First, note that: the man must start and end at vertex ${v_1}$.

### Our methodology

For NP-hard problems, we have three approaches to solve the problem, specifically, (1) exact algorithms, (2) approximation algorithms, and (3) heuristic (or metaheuristic) algorithms:

- The exact approaches find the optimal solution. However, they are exponential time algorithms in the worst case.
- An $\alpha$-approximation algorithm generates a solution that has a factor of $\alpha$ of the optimal solution.

- Heuristic (metaheuristic) algorithms perform well in practice and validate their performance through experiments. This approach is suitable for a problem with large sizes.

Previously, several metaheuristics have been proposed to solve the TSPTW (Dumas, Desrosiers & Gélinas, 1995; Focacci, Lodi & Milano, 2002; Gendreau et al., 1998; Ohlmann & Thomas, 2007; Yuan et al., 2016) and the TRPTW (Ban & Nghia, 2017; Ban, 2021; Heilporna, Cordeaua & Laporte, 2010; Tsitsiklis, 1992). However, they are developed to solve each problem independently and separately. Therefore, they cannot solve both two problems well simultaneously. When we run the two best algorithms for two tasks independently, there is no transferrable knowledge between tasks, and we cannot improve solution quality.

This article proposes an MFEA approach to solve two problems simultaneously. Our MFEA solves two tasks simultaneously: the first task is the TRPTW, and the second is the TSPTW. Experiment results indicate its efficiency: (1) for small instances, the proposed algorithm obtains the optimal solutions in both two problems; (2) for large ones, our solutions are close to the optimal ones, even much better than those of the previous MFEA approaches.

### The MFEA framework introduction

The overview of multifactorial optimization is introduced in Dash et al. (2012) and Gupta, Ong & Feng (2016). Assume that *k* optimization problems are needed to be performed simultaneously. Without loss of generality, tasks are assumed to be minimization problems. The $j$-th task, denoted ${T_i}$, has objective function ${f_j}\!\!:{X_j} \Rightarrow R$, in which ${x_j}$ is solution space. We need to be found $k$ solutions $\{ {x_1},{x_2},...,{x_{k - 1}},{x_k}\} = \min \{ {f_1}(x),{f_2}(x),....,{f_{k - 1}}(x),{f_k}(x)\}$, where ${x_j}$ is a feasible solution in ${X_j}$. Each ${f_j}$ is considered an additional factor that impacts the evolution of a single population of individuals. Therefore, the problem also is called the $k -$ factorial problem. For the problem, a general method to compare individuals is important. Each individual ${p_i}(i \in \{ 1,2,...,|P|\} )$ in a population *P* has a set of properties as follows: Factorial Cost, Factorial Rank, Scalar Fitness, and Skill Factor. These properties allow us to sort and select individuals in the population.

- Factorial Cost $c_j^i$ of the individual ${p_i}$ is its fitness value for task ${T_j}$ $(1 \le j \le k)$.
- Factorial rank $r_j^i$ of ${p_i}$ on the task ${T_j}$ is the index in the set of individuals sorted in ascending order in terms of $c_j^i$.
- Scalar-fitness ${\phi _i}$ of ${p_i}$ is given by its best factorial rank overall tasks as ${\phi _i} = \textstyle{1 \over {{{\min }_{j \in 1,...,k}}r_j^i}}$.
- Skill-factor ${\rho _i}$ of ${p_i}$ is the one task, amongst all other tasks, on which the individual is most effective, *i.e*., ${\rho _i} = argmi{n_j}\{ r_j^i\}$ where $j \in \{ 1,2,...,k\}$.

The pseudo-code of the basic MFEA is described in Algorithm 1 (Ban & Pham, 2022): We first build the unified search space that encompasses all individual search spaces of different tasks to have a shared background on which the transfer of information can take place. We then initialize *SP* individuals (*SP* is the size of population) in the unified search space and then evaluate it by calculating the skill-factor of each individual. After the initialization, the iteration begins to produce the offsprings and assign skill-factors to them. Selective evaluation guarantees that the skill-factor of each new offspring is selected randomly among those of the parents. The offspring and the parent are merged in a new population with $2 \times SP$ individuals. The evaluation for each individual is taken only on the assigned task (in the step, the best solution for each task is updated if it is found. This best solution for each task is the output). After evaluation, the individuals of the population receive new skill-factors. The Elitist strategy keeps the *SP* solutions with the best skill-factors for the next generation.

**Algorithm 1:** The basic MFEA.

1: Build Unified Search Space-USS;
2: Generate a population $p;$
3: Evaluate skill-factor and scalar-fitness in $P;$
4: **while** the stop criteria is not satisfied **do**
5: $O \leftarrow \phi ;$
6: **while** $\left| O \right| \le N$ **do**
7: Sample two individuals ${p_a}$ and ${p_b}$ randomly from top half of parent population;
8: **if** ${\tau _a} = = {\tau _b}$ **then**
9: ${o_a},\,{o_b} \leftarrow$ Intra-task crossover on ${p_a}$ and ${p_b}$
10: Assign offspring ${o_a}$ and ${o_b}$ skill factor ${\tau _a}$
11: **else if** $rand < rmp$ **then**
12: ${o_a},\,{o_b} \leftarrow$ Intra-task crossover on ${p_a}$ and ${p_b}$
13: Randomly assign offspring ${o_a},{o_b}$ skill factor ${\tau _a}$ or ${\tau _b}$
14: **else**
15: ${o_a} \leftarrow$ mutation $({p_a});$
16: ${o_b} \leftarrow$ mutation $({p_b});$
17: ${o_a},\,{o_b}$ have the same skill-factor as ${p_a}$ and ${p_b}$, respectively;
18: $O = O \cup \,\{ {o_a},\,{o_b}\} ;$
19: $p = p \cup \,\{ O\} ;$
20: Evaluate skill-factor and scalar-fitness in $P;$
21: Elitism-Selection $(P);$

**Figure 1:**
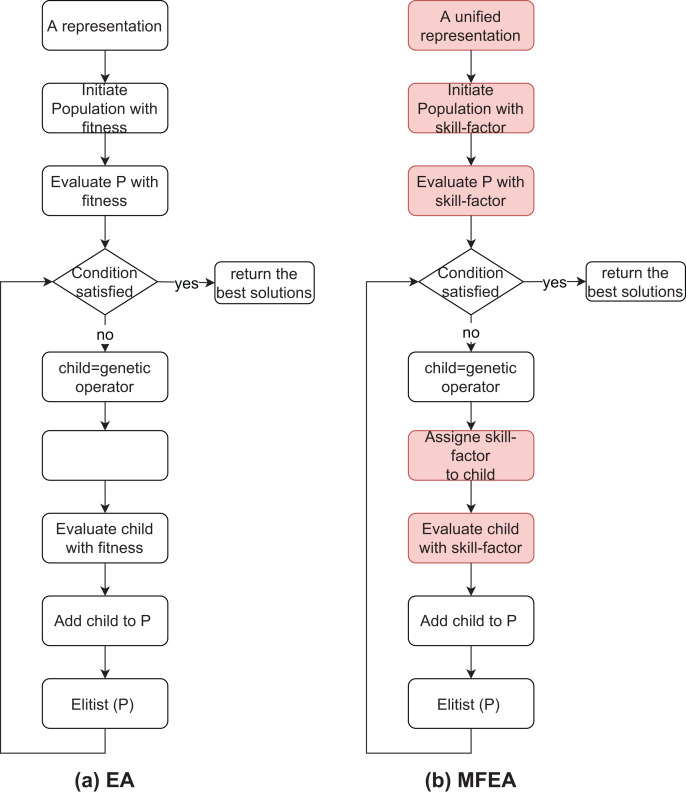
The similarity and difference between EA and MFEA.

Figure 1 (Ban & Pham, 2022) also shows the differences between the traditional EA and MFEA. The crossover and mutation operators in the MFEA are like the traditional EA. However, there are two different important aspects: (1) the parents’ skill-factor and (2) random mating probability (*rmp*). Specifically, the child is created using crossover from parents with the same skill-factor. Otherwise, the child is generated by a crossover with a *rmp* value or by a mutation when parents own different skill-factors. A large *rmp* value generates more information exchanging between tasks. Also, in the traditional GA, the fitness of child is evaluated directly, while the skill-factor is assigned to it in the MFEA.

The MFEA is also unlike multiobjective optimization. In multiobjective optimization, we have one problem with many objective functions. On the other hand, the MFEA solves many tasks at the same time. In addition, multiobjective optimization generally uses a single representative space, while the MFEA unifies multiple representative spaces for many tasks.

Running two algorithms for two tasks independently is not the idea of the MFEA approach. When two algorithms run independently, each task is represented by its own search space. There is no transferrable knowledge between tasks. Otherwise, in the MFEA, two tasks use the unified search space, and transferrable knowledge between tasks is done. It can increase convergence and improve the quality of solutions for multitasks. Lian et al. (2019) then provided a novel theoretical analysis and evidence of the efficiency of the MFEA. This study theoretically explains why some existing the MFEAs perform better than traditional EAs. In addition, the MFEA also can be useful in a system with limited computation.

## The proposed algorithm

**Figure 2:**
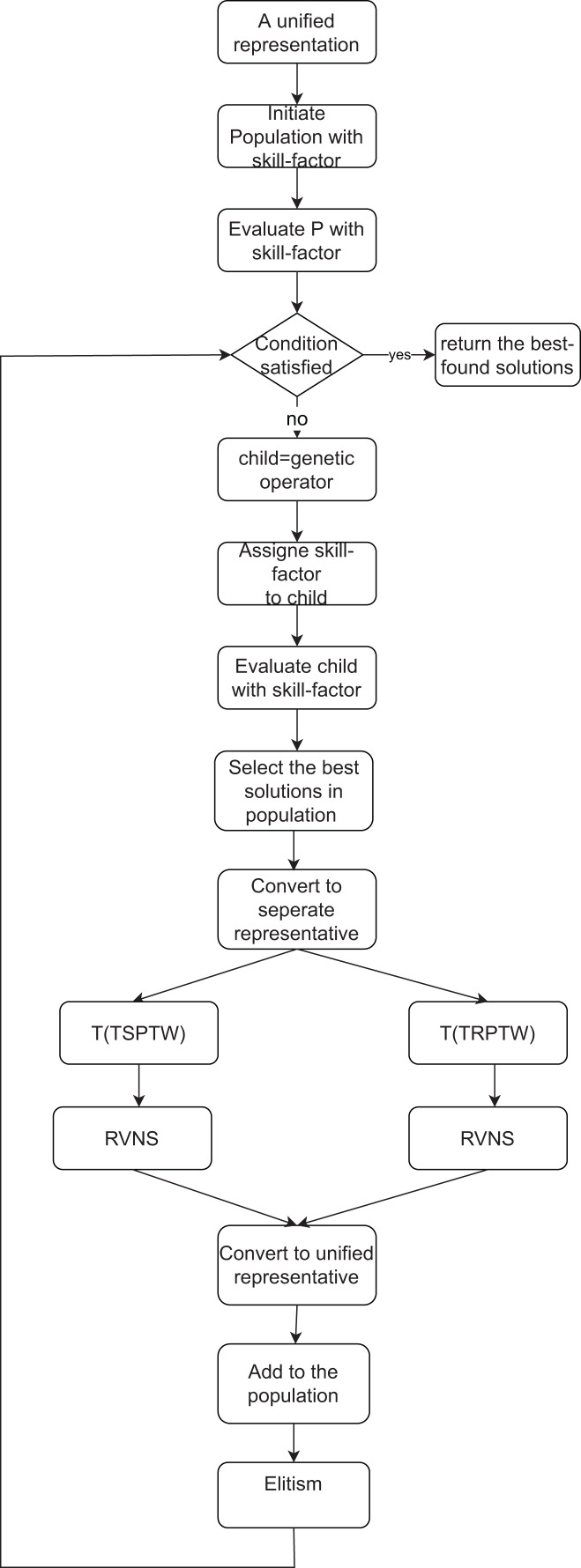
The flow of the proposed MFEA+RVNS.

This section introduces the pseudocode of the proposed MFEA+RNVS. The TSPTW task corresponds to a particular task in the MFEA, while another is the TRPTW task. The flow of the proposed algorithm is described in Fig. 2. Our MFEA+RNVS has core components: unified representation, assortative mating (crossover and mutation operators), selective evaluation, scalar-fitness-based selection, RVNS, and Elitism. The detail of the algorithm is shown in Algorithm 2. More specifically, the algorithm includes the following steps. In the first step, a unified search space is created for both two problems. The population with *SP* individuals is then generated in the second step. All solutions for the population must be feasible. After that, the iteration begins until the termination criterion is satisfied. Parents are selected to produce offsprings using crossover or mutation and then assign skill-factors to them. The offsprings are then added to the current population. The individuals of the population are evaluated to update their scalar-fitness and skill-factor. We select the best solutions regarding skill-factor from the current population and convert them from the unified representation to each task’s one. It is then fed into the RVNS step to find the best solution for each task. The output of the RVNS is then converted to the unified search space. Finally, it is added to the population. The Elitist strategy keeps the *SP* solutions with the best skill-factors for the next generation.

**Algorithm 2:** MFEA+RVNS.

**Require:** ${K_n},{C_{ij}},{v_1},SP$ are the graph, the cost matrix, the starting vertex, and the size of the population.
**Ensure:** The best solution $T_{TSPTW}^*,T_{TRPTW}^*.$
1: $T_{TSPTW}^*,T_{TRPTW}^* \to Inf;$ {Initiate the best solution for the TSPTW, TRPTW}
2: *P* = Construction $({v_1},V,k,\alpha ,level);$ {Initiate the populatin}
3: **while** (The termination criterion of the MFEA is not satisfied) **do**
4: {MFEA step (exploration)}
5: **for** $(j = 1;j\,{\rm {\rm {\leqslant}} }\,SP;j + + )$**do**
6: (*P, M* ) = Selection(*P, NG*); {select parents to mate}
7: **if** (*M* and *P* have the same skill-factor) or (rand(1) $\ {\leqslant}\ $*rmp*) **then**
8: **if** (*M* and *P* have the same skill-factor) **then**
9: ${C_1},{C_2} = {\rm Crossover(}P,M);$
10: ${C_1},{C_2}$’s skill-factors are set to the skill-factors off *P* or *M*, respectively;
11: **else if** (rand(1) ${\leqslant}$ *rmp*) **then**
12: ${C_1},{C_2} = {\rm Crossover(}P,M);$
13: ${C_1},{C_2}$’s skill-factors are set to the skill-factors off *P* or *M* randomly;
14: **if** $({C_1}\,{\rm or}\,{C_2}\,{\rm is\ infeasible)}$**then**
15: **if** ${C_1}$ is infeasible **then**
16: ${C_1}$ = Repair( ${C_1}$); {convert it to feasible one}
17: **if** ${C_2}$ is infeasible **then**
18: ${C_2}$ = Repair( ${C_2}$); {convert it to feasible one}
19: **else**
20: ${C_1}$ = Mutate(*P*);
21: ${C_2}$ = Mutate(*M*);
22: **if** $({C_1}\,{\rm or}\,{C_2}\,{\rm is infeasible)}$ **then**
23: ** if** ${C_1}$ is infeasible **then**
24: ${C_1}$ = Repair( ${C_1}$); {convert it to feasible one}
25: **if** ${C_2}$ is infeasible **then**
26: ${C_2}$ = Repair( ${C_2}$); {convert it to feasible one}
27: ${C_1}$’s, ${C_2}$’s skill-factor is set to *P*, *M*, respectively;
28: $P = P \cup \{ {C_1},{C_2}\} ;$
29: Update scalar-fitness and skill-factor for all individuals in *P*;
30: *LT* = Select the best individuals from *P*;
31: {RVNS step (exploitation)}
32: **for** each *T* in *LT* **do**
33: $({T_{TSPTW}},{T_{TRPTW}})$ = Convert *T* from unified representation to one for each task;
34: $T_{TSPTW}^{\rm '} = {\rm RVNS}({T_{TSPTW}});$ {local search}
35: **if** $(T_{TSPTW}^{'} < T_{TSPTW}^*)$ **then**
36: $T_{TSPTW}^{'} \to T_{TSPTW}^*;$
37: $T_{TSPTW}^{\rm '} = {\rm RVNS}({T_{TSPTW}});$ {local search}
38: **if** $(T_{TSPTW}^{'} < T_{TSPTW}^*)$ **then**
39: $T_{TSPTW}^{'} \to T_{TSPTW}^*;$
40: ${T^{'}} = {\rm convert}(T_{TSPTW}^{'},T_{TRPTW}^{'})$ to unified representation;
41: $P = P \cup \{ {T^{'}}\} ;$
42: *P* = Elitism-Selection(*P*); {keep the best *SP* individuals}
43: **return** $T_{TSPTW}^*,T_{TRPTW}^*;$

### Creating unified search space-USS

**Figure 3:**
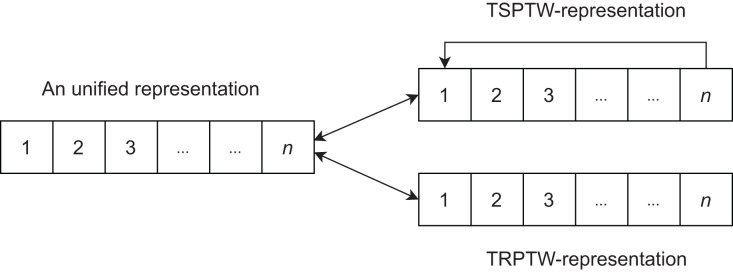
The interpretation of unified representation for each task.

In the literature, various representations are proposed for two problems. Among these representations, the permutation representation shows efficiency compared to the others. In the permutation, each individual is coded as a set of $n$ vertices $({v_1},{v_2},...,{v_k},...,{v_n})$, where $k$ is the $k -$th index. Figure 3 demonstrates the encoding for two problems.

### Initializing population

Each feasible solution is created from the RVNS to take a role as an individual in the population. Therefore, we have *S*_*p*_ individuals in the initial population for the genetic step.

Algorithm 3 describes the constructive step. The objective function is the sum of all positive differences between the arrival time $(D({v_i}))$ and its due time $({l_i})$ on each vertex. Specifically, it is $\min \sum\limits_{i = 1}^n {\max } (0,D({v_i}) - {l_i})$. The algorithm runs until it finds a feasible solution. Restricted Candidate List (*RCL*) is created by ordering all non-selected vertices based on a greedy function that evaluates the benefit of including them in the solution. One vertex is then chosen from *RCL* randomly. Since all vertices are visited, we receive a solution. If this solution is a feasible one, it is an initial solution, and this step stops. Conversely, a repair procedure based on the RVNS with many neighborhoods (Johnson & McGeoch, 2003) is invoked, and the procedure iterates until a feasible solution is reached. The solution is shaken to escape from the current local optimal solution. The RVNS is then applied to create the new solution. If it is better than the best-found solution, it is set to the new current solution. The *level* is increased by one if the current solution is not improved, or reset to 1, otherwise. The repair procedure is described in Algorithm 4.

**Algorithm 3:** Construction.

**Require:** ${v_1},V,k,\alpha ,$ *level* are a starting vertex, the set of vertices in ${K_n}$, the number of vehicles and the size of *RCL*, the parameter to control the strength of the perturbation procedure, respectively.
**Ensure:** An initial solution *T*.
1: $P = \phi ;$ {Initially, the population is empty}
2: **while ** $(\left| P \right| < SP)$ **do**
3: $T = \{ {v_1}\} ;$ {*T* is a tour and it starts at ${v_1}$}
4: **while** $\left| T \right| < n$ **do**
5: Create *RCL* that includes $\alpha$ nearest vertices to ${v_e}$ in *V*; { ${v_e}$ is the last vertex in *T*}
6: Select randomly vertex $v = \{ {v_i}|{v_i} \in RCL\,{\rm \ and }\;{v_i}\ \notin\ T\} ;$
7: $T \leftarrow T \cup \{ {v_i}\};$ {add the vertex to the tour}
8: **if** *T* is infeasible solution **then**
9: {Convert infeasible solution to feasible one}
10: *T* = Repair (*T*, *level_max*, ${N_i}$(*i* = 1,…,7));
11: $P = P \cup \{ {T^{'}}\}$; {add the tour to the population}
12: return *P*;

**Algorithm 4:** Repair (*T level_max*, ${N_i}$(*I* = 1,…,7)).

**Require:** *T, level_max*, ${N_i}$(*i* = 1,…,7) are the infeasible solution, the parameter to control the strength of the perturbation procedure, and the number of neighbourhood respectively.
**Ensure:** An feasible solution *T*.
1: *level* = 1;
2: **while** ((*T* is infeasible solution) and (*level* $\le$ *level_max))* **do**
3: ${T^{'}}$= Perturbation(*T, level*);
4: **for** $i:1 \to 6$ **do**
5: ${T^{''}} \leftarrow {\rm arg}\min {N_i}({T^{'}});$ {local search}
6: **if** $(L({T^{''}} < L({T^{'}}))$ **then**
7: ${T^{'}} \leftarrow {T^{''}}$
8: $i \leftarrow 1$
9: **else**
10: $i + +$
11: **if** $L({T^{'}}) < L(T)$ **then**
12: $T \leftarrow {T^{'}};$
13: **if** $L({T^{'}}) = = L(T)$ **then**
14: $level \leftarrow 1;$
15: **else**
16: $level + + ;$
17: return *T*;

In this article, some neighborhoods widely applied in the literature (Johnson & McGeoch, 2003). We describe more details about seven neighborhoods as follows:

- **move** moves a vertex forward one position in *T*.
- **shift** relocates a vertex to another position in *T*.
- **swap-adjacent** attempts to swap each pair of adjacent vertices in the tour.
- **exchange** tries to swap the positions of each pair of vertices in the tour.
- **2-opt** removes each pair of edges from the tour and reconnects them.
- **Or-opt**: Three adjacent vertices are reallocated to another position of the tour.

### Evaluating for individuals

The scalar-fitness function demonstrates the way of evaluating individuals. Scalar-fitness then are calculated for each individual. The larger and larger the scalar-fitness value is, the better and better the individual is.

### Selection operator

In the original tournament (Ban & Pham, 2022; Talbi, 2009), the fitness is the only criterion in choosing parents. This article adapts this selection for the MFEA algorithm balancing scalar-fitness and population diversity. It means the selection mechanism simultaneously promoting both diversity and scalar-fitness. For each solution, we count its scalar-fitness and its diversity in a set of solutions as follows:

(1) $$R(T) = (1 - \alpha ) \times (SP - RF(T) + 1) + \alpha \times (SP - RD(T) + 1)$$

where *SP*, $\alpha \in [0,1]$, $RF(T)$, and $RD(T)$ are the population size, threshold, the rank of *T* in the *P* based on the scalar-fitness, and the rank of *T* in the *P* based on its diversity, respectively.

(2) $$\bar d(T) = \displaystyle{{\sum\nolimits_{i = 1}^n d (T,{T_i})} \over n}$$

The metric distance between two solutions is the minimum number of transformations from one to another. We define the distance $d(T,{T_i})$ to be *n* (the number of vertices) minus the number of vertices with the same position on *T* and ${T_i}$. Similarly, $\overline {d(T)}$ is the average distance of *T* in the population. The larger $\bar d(T)$ is, the higher its rank is. The larger *R* is, the better solution *T* is.

The selection operator selects individual parents based on their *R* values to mate. We choose the tournament selection operator (Talbi, 2009) because of its efficiency. A group of *NG* individuals is selected randomly from the population. Then, two individuals with the best *R* values in the group are chosen to become parents. The selection pressure can be increased by extending the size of the group. On average, the selected individuals from a larger group have higher *R* values than those of a small size. The detail in this step is described in Algorithm 5.

**Algorithm 5:** Selection operator (P, NG).

**Require:** *P, NG* are the population and the size of group, respectively.
**Ensure:** Parents ${C_1} , {C_2}$.
1: Select randomly the NG individuals in the *P*;
2: Sort them in terms of their *R* values;
3: ${C_1} , {C_2}$ = Select two individuals with the best *R* values;
4: return ${C_1} , {C_2}$;

### Crossover operator

The crossover is implemented with the predefined probability (*rmp*) or if the parents have the same skill-factor. When parents have the same skill-factor, we have inter-crossover. Otherwise, the intra-crossover is applied to parents with different skill-factor. It opens up the chance for knowledge transfer by using crossover-based exchange between tasks. In Otman & Jaafar (2011), the crossovers are divided into three main types. We found no logical investigation showing which operator brings the best performance in the literature. In a preliminary study, we realize that the algorithm’s effectiveness relatively depends on selected crossover operators. Since trying all operators leads to computationally expensive efforts, our numerical analysis is conducted on randomly selected operators for each type. The following operators are selected from the study to balance solution quality and running time.

- The first type is related to the position of certain genes in parents (PMX, CX).
- The second selects genes alternately from both parents, without genes’ repetition (EXX, EAX).
- The third is an order-based crossover (SC, MC).

Initially, we select a crossover randomly. If any improvement of the best solution is found, the current crossover operator is continued to use. Otherwise, if the improvement of the best solution is not found after the number of generations (*NO*), another crossover operator is replaced randomly. Using multiple crossovers helps the population be more diverse than one crossover. Therefore, these operators prevent the algorithm from premature convergence. If the offsprings are infeasible, the fix procedure is invoked to convert them to feasible ones. The offsprings’ skill-factors are randomly set to the one of the father or mother. The detail in this step is described in Algorithm 6.

**Algorithm 6:** Crossover (P, M).

**Require:** *P, M* are the parent tour, respectively.
**Ensure:** A new child *T*.
1*: type* = rand (3);
2: *rnd* = rand (2);
3: **if** (*type == 1*) **then**
4: {the first type crossover is selected}
5: **if** (*rnd == 1*) **then**
6: *C* = **PMX** (*P, M*); {PMX is chosen}
7: **else if** (*rnd==2*) **then**
8: *C* = **CX** (*P, M*); {CX is selected}
9: **else if** (*type==2*) **then**
10: {the second type is selected}
11: **if** (*rnd == 1*) **then**
12: *C* = **EXX** (*P, M*); {EXX is selected}
13: **else if** (*rnd==2*) **then**
14: *C* = **EAX** (*P, M*); {EAX is selected}
15: **else if** (*type==3*) **then**
16: {the type 3 is selected}
17: **if** (*rnd == 1*) **then**
18: *C* = **SC** (*P, M*); {SC is selected}
19: **else if** (*rnd==2*) **then**
20: *C* = **MC** (*P, M*); {MC is selected}

### Mutation operator

A mutation is used to keep the diversity of the population. Some mutations are used in the proposed algorithm:

- The Inversion Mutation picks two vertices at random and then inverts the list of vertices between them. It preserves most adjacency information and only breaks two links, leading to the disruption of order information.
- The Insertion Mutation removes the vertex from the current index and then inserts it in a random index on the solution. The operator preserves most of the order and the adjacency information.
- Swap Mutation selects two vertices at random and swaps their positions.

It preserves most of the adjacency information, but links broken disrupt order more. We randomly select one of three operators when this mutation is performed. After the mutation operator, two offsprings are created from the parents. If the offsprings are infeasible, the repair procedure converts them to feasible ones. Their skill-factors are set to those of parents, respectively. The detail in the mutation is described in Algorithm 7.

**Algorithm 7:** Mutate (*C*).

**Require:** *C* is the child, respectively.
**Ensure:** A new child *C*.
1: {Choose a mutation operator randomly}
2: *rnd* = rand (2);
3: **if** (*rnd == 1*) **then**
4: *C* = **Inversion(C)** {Inversion mutation is selected}
5: **else if** (*rnd==2*) **then**
6: *C* = **Insertion(C)** {Inversion mutation is selected}
7: **else**
8: *C* = **Swap(C)** {Swap mutation is selected}
9: return *C*;

### RVNS

The combination between the MFEA and the RVNS allows good transferrable knowledge between tasks from the MFEA and the ability to exploit good solution spaces from RVNS. We select some best solutions in the current population to feed into the RVNS. In the RVNS step, we convert a solution from unified representation to separated representation for each task. The RVNS then applies to each task separately. Finally, the output of the RVNS is represented in the unified space. The improved solution will be added to the population.

For this step, we use popular neighborhoods such as move, shift, swap-adjacent, exchange, 2-opt, and or-opt in Johnson & McGeoch (2003) and Reeves (1999). In addition, the pseudocode of the RVNS algorithm is given in Algorithm 8.

**Algorithm 8:** RVNS (T).

**Require:** *T* is a tour.
**Ensure:** A new solution *C*.
1: Initialize the Neighbourhood List *NL*;
2: **while** *NL* $\ne$ 0 **do**
3: Choose a neighbourhood *N* in *NL* at random
4: ${T^{'}}\, \leftarrow \,\arg \,\min \,N(T)\,;$ {Neighbourhood search}
5: **if** $((W({T^{'}})\, < \,W(T))\,\,$ and $({T^{'}}\,{\rm is feasible))}$ **then**
6: $T \leftarrow \,{T^{'}}$
7: Update *NL*;
8: **else**
9: Remove *N* from the *NL*;
10: **if** $((W({T^{'}})\, < \,W({T^*}))\,\,$ and $({T^{'}}\,{\rm is feasible))}$ **then**
11: ${T^*} \leftarrow \,{T^{'}}$

### Elitism operator

Elitism is a process that ensures the survival of the fittest, so they do not die through the evolutionary processes. Researchers show the number (Talbi, 2009) (usually below 15%) of the best solutions that automatically go to the next generation. The proposed algorithm selects *Sp* individuals for the next generation, in which about 15% of them are the best solutions in the previous generation, and the remaining individuals are chosen randomly from *P*.

### The stop condition

After the number of generations $(Ng)$, if the best solution has not been improved, then the proposed algorithm stops.

## Computational evaluvations

The experiments are conducted on a personal computer equipped with a Xeon E-2234 CPU and 16 GB bytes of RAM. The program was coded in C $\ne$ language. The generation number (*Ng*), population size (*SP*), group size (*NG*) in the selection, and crossover rates (*rmp*) influence the algorithm’s results. Many efforts in the literature studied the algorithm sensitivity to parameter changes. We found that no work shows which values are the best for all cases. However, the following suggestions help us in choosing parameter values:

- A large generation number does not improve performance. Besides, it consumes much time to run. A small value makes the algorithm fail to reach the best solution (Angelova & Pencheva, 2011).
- A higher crossover value obtains new individuals more quickly while a low crossover rate may cause stagnation (Angelova & Pencheva, 2011).
- A large population size can increase the population diversity. However, it can be unhelpful in the algorithm and increase the running time of it (Chen et al., 2012).
- Increased selection pressure can be provided by simply increasing the group size. When the selection pressure is too low, the convergence rate is slower, while if it is too high, the chance of the algorithm prematurely converges (Lavinas et al., 2019).
- The $\alpha$ and level values help to create the diversity of the initial population. A larger value leads to the same as the random method, while a small value decreases the diversity.

Based on the suggestions, we determine a suitable range for each parameter in Table 1. In the next step, we choose the best value from the range as follows: finding the best configuration by conducting all instances would have been too expensive in computation, and we test numerical analysis on some instances. The configuration selected in many combinations is tested, and the one that has obtained the best solution is chosen. In Table 1, we determine a range for each parameter that generates different combinations, and we run the proposed algorithm on some selected instances of the combinations. We find the following settings so that our algorithm obtains the best solutions: $SP = 100,NG = 5,rmp = 0.7,\alpha = 10,level = 5$, and $Ng = 100$. This parameter setting has thus been used in the following experiments.

**Table 1:** The variable parameters.

Parameter	Value range
*SP*	$50 \le {\beta _r} \le 200$, incremented by 50
*NG*	$5 \le \alpha \le 15$, incremented by 5
*rmp*	$0.5 \le {\beta _\eta } \le 1$, incremented by 0.1
$\alpha$	$5 \le {\tau _0} \le 20$, incremented by 5
*level*	$5 \le p \le 15$, incremented by 5
*Ng*	$50 \le Ng \le 150$, incremeted by 50

We found no algorithm based on the literature’s MFEA framework for the TRPTW and TSPTW. Therefore, the proposed algorithm’s results directly compare with the known best solutions of the TSPTW and TRPTW on the same benchmark. Moreover, to compare with the previous MFEA framework (Osaba et al., 2020; Yuan et al., 2016), our MEFA+RVNS is tested on the benchmark for the TSP and TRP. They are specific variants of TSPTW and TRPTW without time window constraints. Therefore, the instances are used in the article as follows:

- Dumas, Desrosiers & Gélinas (1995) propose the first set citebib09 and contains 135 instances grouped in 27 test cases. Each group has five Euclidean instances, coordinates between 0 and 50, with the same number of customers and the same maximum range of time windows. For example, the instances n20w60.001, n20w60.002, n20w60.003, n20w60.004, and n20w60.005 have 20 vertices and the time window for each vertex is uniformly random, between 0 and 60.
- Gendreau et al. (1998) propose the second set of instances citebib12 and contains 140 instances grouped in 28 test cases.
- Ohlmann & Thomas (2007) propose the third set of instances citebib30 and contains 25 instances grouped in five test cases.
- The fourth sets in the majority are the instances proposed by Dumas, Desrosiers & Gélinas (1995) with wider time windows.
- The TSPLIB (http://comopt.ifi.uni-heidelberg.de/software/TSPLIB95/) includes fourteen instances from 50 to 100 instances.

The efficiency of the metaheuristic algorithm can be evaluated by comparing the best solution found by the proposed algorithm (notation: *Best.Sol*) to (1) the optimal solution (notation: *OPT*); and (2) the known best solution (notation: *KBS*) of the previous metaheuristics (note that: In the TSPTW, *KBS* is the optimal solution) as follows:

(3) $$gap[\% ] = \displaystyle{{Best.Sol - KBS(OPT)} \over {KBS(OPT)}} \times 100\%$$

The smaller and smaller the value of *gap* is, the better and better our solution is. All instances and found solutions are available in the link https://sites.google.com/soict.hust.edu.vn/mfea-tsptw-trptw/home.

In Tables, *OPT*, *Aver.Sol* and *Best.Sol* are the optimal, average, and best solution after ten runs, respectively. Let *Time* be the running time such that the proposed algorithm reaches the best solution. Note that: Yuan et al. (2016) supported the source code of their algorithm in Yuan et al. (2016) while the dMFEA-II (dMFEA-II is the MFEA with dynamic *rmp* value) (Osaba et al., 2020) was implemented again by us. Tables 2 and 3 evaluate the efficiency of the proposed selection in MFEA+RVNS. Tables 4–8 compare the proposed MFEA+RVNS with the known best or optimal solutions for the TSPTW and TRPTW instances (Abeledo et al., 2013; Ban & Nghia, 2017; Ban, 2021; Dumas, Desrosiers & Gélinas, 1995; Heilporna, Cordeaua & Laporte, 2010; Silva & Urrutia, 2010; Ohlmann & Thomas, 2007; Salvesbergh, 1985; Reeves, 1999). In the Tables, the *KBS*, *OPT*, *Aver.Sol*, and *Best.Sol* columns are the best known, optimal, average, and best solution, respectively, while the *gap* column presents the difference between the best solution and the optimal one. Table 9 shows the average values of Tables 4–7 comparing the MFEA+RVNS, OA (Osaba et al., 2020), and YA (Yuan et al., 2016). In the TSP, the optimal solutions of the TSPLIB-instances are obtained by running the Concord tool (https://www.math.uwaterloo.ca/tsp/concorde.html). The optimal or best solutions in the TRP are obtained from Abeledo et al. (2013).

**Table 2:** Comparison the best-found values between MFEA-NR and MFEA for TSPTW and TRPTW instances proposed by Dumas, Desrosiers & Gélinas (1995), and Silva & Urrutia (2010).

Instances	MFEA-NR		MFEA+RNVS		diff[%]	
	TSPTW	TRPTW	TSPTW	TRPTW	TSPTW	TRPTW
n20w20.002	286	2,560	286	2,560	0.00	0.00
n20w40.002	333	2,679	333	2,679	0.00	0.00
n20w60.002	244	2,176	244	2,176	0.00	0.00
n20w80.003	338	2,669	338	2,669	0.00	0.00
n20w100.002	222	2,082	222	2,082	0.00	0.00
n40w40.002	483	7,202	461	7,104	−4.55	−1.36
n40w60.002	487	7,303	470	7,247	−3.49	−0.77
n40w80.002	468	7,209	431	7,123	−7.91	−1.19
n40w100.002	378	6,789	364	6,693	−3.70	−1.41
n60w20.002	626	14,003	605	13,996	−3.35	−0.05
n60w120.002	472	12,622	427	12,525	−9.53	−0.77
n60w140.002	475	11,914	464	11,810	−2.32	−0.87
n60w160.002	443	12,920	423	12,719	−4.51	−1.56
n80w120.002	587	18,449	577	18,383	−1.70	−0.36
n80w140.002	495	18,243	472	18,208	−4.65	−0.19
n80w160.002	588	17,334	553	17,200	−5.95	−0.77
aver					−3.23	−0.58

**Table 3:** Comparison the best-found values between MFEA-NLS and MFEA for TSPTW and TRPTW instances proposed by Dumas, Desrosiers & Gélinas (1995), and Silva & Urrutia (2010).

Instances	MFEA-NLS		MFEA+RNVS		diff[%]	
	TSPTW	TRPTW	TSPTW	TRPTW	TSPTW	TRPTW
n20w20.002	286	2,560	286	2,560	0.00	0.00
n20w40.002	333	2,679	333	2,679	0.00	0.00
n20w60.002	244	2,176	244	2,176	0.00	0.00
n20w80.003	338	2,669	338	2,669	0.00	0.00
n20w100.002	222	2,082	222	2,082	0.00	0.00
n40w40.002	522	7,530	461	7,104	−11.69	−5.66
n40w60.002	503	7,517	470	7,247	−6.56	−3.59
n40w80.002	475	7,763	431	7,123	−9.26	−8.24
n40w100.002	400	7,502	364	6,693	−9.00	−10.78
n60w20.002	626	14,097	605	13,996	−3.35	−0.72
n60w120.002	480	13,680	427	12,525	−11.04	−8.44
n60w140.002	502	12,951	464	11,810	−7.57	−8.81
n60w160.002	461	13,953	423	12,719	−8.24	−8.84
n80w120.002	620	19,860	577	18,383	−6.94	−7.44
n80w140.002	520	19,742	472	18,208	−9.23	−7.77
n80w160.002	614	19,516	553	17,200	−9.93	−11.87
aver					−5.80	−5.14

**Table 4:** Comparison between our results with the best-found values for TSPTW and TRPTW instances proposed by Dumas, Desrosiers & Gélinas (1995), and Silva & Urrutia (2010).

Instances	TSPTW	TRPTW	MFEA+RNVS							
			TSPTW				TRPTW			
	*OPT*	*KBS*	*Best.Sol*	*Aver.Sol*	*gap*	*Time*	*Best.Sol*	*Aver.Sol*	*gap*	*Time*
n20w20.001	378	2,528	378	378	0.0	3	2,528	2,528	0.0	3
n20w20.002	286	2,560	286	286	0.0	2	2,560	2,560	0.0	2
n20w20.003	394	2,671	394	394	0.0	2	2,671	2,671	0.0	2
n20w20.004	396	2,975	396	396	0.0	6	2,975	2,975	0.0	6
n20w40.001	254	2,270	254	254	0.0	2	2,270	2,270	0.0	2
n20w40.002	333	2,679	333	333	0.0	5	2,679	2,679	0.0	5
n20w40.003	317	2,774	317	317	0.0	3	2,774	2,774	0.0	2
n20w40.004	388	2,568	388	388	0.0	2	2,568	2,568	0.0	3
n20w60.001	335	2,421	335	335	0.0	3	2,421	2,421	0.0	2
n20w60.002	244	2,176	244	244	0.0	2	2,176	2,176	0.0	2
n20w60.003	352	2,694	352	352	0.0	2	2,694	2,694	0.0	2
n20w60.004	280	2,020	280	280	0.0	2	2,020	2,020	0.0	2
n20w80.001	329	2,990	329	329	0.0	2	2,990	2,990	0.0	3
n20w80.002	338	2,669	340	340	0.6	1	2,669	2,669	0.0	2
n20w80.003	320	2,643	320	320	0.0	2	2,643	2,643	0.0	2
n20w80.004	304	2,627	306	306	0.7	3	2,552	2,552	−2.9	2
n20w100.001	237	2,294	237	237	0.0	3	2,269	2,269	−1.1	3
n20w100.002	222	2,082	222	222	0.0	2	2,082	2,082	0.0	2
n20w100.003	310	2,416	310	310	0.0	2	2,416	2,416	0.0	3
n20w100.004	349	2,914	349	349	0.0	1	2,862	2,862	−1.8	2
n40w20.001	500	7,875	500	500	0.0	9	7,875	7,875	0.0	8
n40w20.002	552	7,527	552	552	0.0	7	7,527	7,527	0.0	8
n40w20.003	478	7,535	478	478	0.0	8	7,535	7,535	0.0	9
n40w20.004	404	7,031	404	404	0.0	8	7,031	7,031	0.0	9
n40w40.001	465	7,663	465	465	0.0	7	7,663	7,663	0.0	9
n40w40.002	461	7,104	461	461	0.0	8	7,104	7,104	0.0	8
n40w40.003	474	7,483	474	474	0.0	8	7,483	7,483	0.0	8
n40w40.004	452	6,917	452	452	0.0	8	6,917	6,917	0.0	9
n40w60.001	494	7,066	494	494	0.0	9	7,066	7,066	0.0	7
n40w60.002	470	7,247	470	470	0.0	8	7,247	7,247	0.0	8
n40w60.003	408	6,758	410	410	0.0	9	6,758	6,758	0.0	8
n40w60.004	382	5,548	382	382	0.0	9	5,548	5,548	0.0	9
n40w80.001	395	8,229	395	395	0.0	8	8,152	8,152	0.0	9
n40w80.002	431	7,176	431	431	0.0	8	7,123	7,123	0.0	9
n40w80.003	412	7,075	418	418	0.0	8	7,075	7,075	0.0	9
n40w80.004	417	7,166	417	417	0.6	9	7,166	7,166	0.0	10
n40w100.001	429	6,858	432	432	0.0	8	68,00	6,800	0.0	9
n40w100.002	358	6,778	364	364	0.7	11	6,693	6,693	−2.9	10
n40w100.003	364	6,178	364	364	0.0	9	6,926	6,926	−1.1	11
n40w100.004	357	7,019	361	361	0.0	9	7,019	7,019	0.0	8
n60w20.002	605	13,996	605	605	0.0	18	13,996	13,996	0.0	19
n60w20.003	533	13,782	533	533	0.0	17	12,965	12,965	−1.8	18
n60w20.004	616	12,965	616	616	0.0	17	15,102	15,102	0.0	18
n60w40.003	603	15,034	612	612	0.0	19	15,034	15,034	0.0	19

**Table 5:** Comparison between our results with the best-found values for TSPTW and TRPTW instances proposed by Dumas, Desrosiers & Gélinas (1995), and Silva & Urrutia (2010).

Instances	TSPTW	TRPTW	MFEA+RNVS							
			TSPTW				TRPTW			
	*OPT/KBS*	*KBS*	*Best.Sol*	*Aver.Sol*	*Gap*	*Time*	*Best.Sol*	*Aver.Sol*	*Gap*	*Time*
n60w160.004	401	11,645	401	401	0.0	19	11,778	11,778	1.1	19
n60w180.002	399	12,015	399	399	0.0	17	12,224	12,224	1.7	21
n60w180.003	445	12,214	445	445	0.0	18	12,679	12,679	3.8	21
n60w180.004	456	11,101	456	456	0.0	19	11,245	11,245	1.3	18
n60w200.002	414	11,748	414	414	0.0	20	11,866	11,866	1.0	19
n60w200.003	455	10,697	460	460	1.1	19	10,697	10,697	0.0	18
n60w200.004	431	11,441	431	431	0.0	16	11,740	11,740	2.6	17
n80w120.002	577	18,181	577	577	0.0	17	18,383	18,383	1.1	21
n80w120.003	540	17,878	548	548	1.5	19	17,937	17,937	0.3	21
n80w120.004	501	17,318	501	501	0.0	18	17,578	17,578	1.5	18
n80w140.002	472	17,815	472	472	0.0	19	18,208	18,208	2.2	21
n80w140.003	580	17,315	580	580	0.0	20	17,358	17,358	0.2	21
n80w140.004	424	18,936	424	424	0.0	19	19,374	19,374	2.3	18
n80w160.002	553	17,091	553	553	0.0	27	17,200	17,200	0.6	18
n80w160.003	521	16,606	521	521	0.0	21	16,521	16,521	−0.5	20
n80w160.004	509	17,804	509	509	0.0	20	17,927	17,927	0.7	18
n80w180.002	479	17,339	479	479	0.0	21	17,904	17,904	3.3	19
n80w180.003	530	17,271	530	530	0.0	20	17,160	17,160	−0.6	20
n80w180.004	479	16,729	479	479	0.0	22	16,849	16,849	0.7	21
n100w120.002	540	29,882	556	556	3.0	38	29,818	29,818	0.0	45
n100w120.003	617	25,275	646	646	4.7	37	24,473	24,473	0.0	42
n100w120.004	663	30,102	663	663	0.0	39	31,554	31,554	0.0	41
n100w140.002	622	30,192	632	632	1.6	38	30,087	30,087	0.0	45
n100w140.003	481	28309	481	481	0.0	39	28,791	28,791	0.0	47
n100w140.004	533	27,448	533	533	0.0	40	27,990	27,990	0.0	45
n150w120.003	747	42,340	769	769	2.9	75	42,339	42,339	0.0	72
n150w140.001	762	42,405	785	785	3.0	70	42,388	42,388	−0.1	74
n150w160.001	706	45,366	732	732	3.6	72	45,160	45,160	−0.4	78
n150w160.002	711	44,123	735	735	3.3	74	44,123	44,123	0.0	76
n200w200.001	9,424	1,094,630	9,424	9,424	0.0	101	1,093,537	1,093,537	−0.1	89
n200w200.002	9,838	1,099,839	9,885	9,885	0.5	110	1,099,839	1,099,839	0.0	86
n200w200.003	9,043	1,067,171	9,135	9,135	1.0	99	1,067,161	1,067,161	0.0	93
n200w300.001	7,656	1,052,884	7,791	7,791	1.7	100	1,047,893	1,047,893	−0.5	106
n200w300.002	7,578	1,047,893	7,721	7,721	1.8	105	1,047,893	1,047,893	0.0	110
n200w300.003	8,600	1,069,169	8,739	8,739	1.6	120	1,069,169	1,069,169	0.0	93
n200w300.004	8,268	1,090,972	8,415	8,415	1.7	112	1,090,972	1,090,972	0.0	96
n200w300.005	8,030	1,022,000	8,190	8,190	1.9	114	1,016,765	1,016,765	−5.1	98
n200w400.001	7,420	1,064,456	7,661	7,661	3.2	109	1,064,456	1,064,456	0.0	100
aver					0.49	26.24			−0.11	25.6

**Table 6:** Comparison between our results with the best-found values for TSPTW and TRPTW instances proposed by Gendreau et al. (1998), and Ohlmann & Thomas (2007).

Instances	TSPTW	TRPTW	MFEA+RNVS							
			TSPTW				TRPTW			
	*KBS*	*KBS*	*Best.Sol*	*Aver.Sol*	*Gap*	*Time*	*Best.Sol*	*Aver.Sol*	*Gap*	*Time*
n20w120.002	218	2,193	218	218	0.0	2	2,193	2,193	0.0	3
n20w120.003	303	2,337	303	303	0.0	4	2,337	2,337	0.0	2
n20w120.004	300	2,686	300	300	0.0	2	2,686	2,686	0.0	2
n20w140.002	272	2,330	272	272	0.0	2	2,330	2,330	0.0	3
n20w140.003	236	2,194	236	236	0.0	2	2,196	2,196	0.1	2
n20w140.004	255	2,279	264	264	3.5	4	2,278	2,278	0.0	5
n20w160.002	201	1,830	201	201	0.0	2	1,830	1,830	0.0	2
n20w160.003	201	2,286	201	201	0.0	3	2,286	2,286	0.0	2
n20w160.004	203	1,616	203	203	0.0	2	1,616	1,616	0.0	2
n20w180.002	265	2,315	265	265	0.0	4	2,315	2,315	0.0	2
n20w180.003	271	2,414	271	271	0.0	2	2,414	2,414	0.0	2
n20w180.004	201	2,624	201	201	0.0	3	1,924	1,924	−26.7	2
n20w200.002	203	1,799	203	203	0.0	2	1,799	1,799	0.0	2
n20w200.003	249	2,144	260	260	4.4	2	2,089	2,089	−2.6	1
n20w200.004	293	2,624	293	293	0.0	1	2,613	2,613	−0.4	2
n40w120.002	445	6,265	446	446	0.2	3	6,265	6,265	0.0	8
n40w120.003	357	6,411	360	360	0.8	2	6,411	6,411	0.0	7
n40w120.004	303	5,855	303	303	0.0	3	5,855	5,855	0.0	6
n40w140.002	383	5,746	383	383	0.0	2	5,746	5,746	0.0	8
n40w140.003	398	6,572	398	398	0.0	3	6,572	6,572	0.0	7
n40w140.004	342	5,719	350	350	2.3	8	5,680	5,680	−0.7	8
n40w160.002	337	6,368	338	338	0.3	9	6,351	6,351	−0.3	8
n40w160.003	346	5,850	346	346	0.0	9	5,850	5,850	0.0	9
n40w160.004	288	4,468	289	289	0.3	8	4,440	4,440	−0.6	9
n40w180.002	347	6,104	349	349	0.6	8	6,104	6,104	0.0	9
n40w180.003	279	6,040	282	282	1.1	7	6,031	6,031	−0.1	8
n40w180.004	354	6,103	361	361	2.0	8	6,283	6,283	2.9	8
n40w200.002	303	6,674	303	303	0.0	8	5,830	5,830	−12.6	9
n40w200.003	339	5,542	343	343	1.2	7	5,230	5,230	−5.6	8
n40w200.004	301	6,103	301	301	0.0	9	5,977	5,977	−2.1	10
n60w120.002	427	12,517	427	427	0.0	19	12,525	12,525	0.1	19
n60w120.003	407	11,690	419	419	2.9	19	11,680	11,680	−0.1	19
n60w120.004	490	11,132	492	492	0.4	13	11,135	11,135	0.0	19
n60w140.002	462	11,782	464	464	0.4	18	11,810	11,810	0.2	19
n60w140.003	427	13,128	448	448	4.9	13	13,031	13,031	−0.7	16
n60w140.004	488	13,189	488	488	0.0	15	12,663	12,663	−4.0	15
n60w160.002	423	12,471	423	423	0.0	19	12,719	12,719	2.0	17
n60w160.003	434	10,682	447	447	3.0	14	10,674	10,674	−0.1	15

**Table 7:** Comparison between our results with the best-found values for TSPTW and TRPTW instances proposed by Gendreau et al. (1998), and Ohlmann & Thomas (2007).

Instances	TSPTW	TRPTW	MFEA+RNVS							
			TSPTW				TRPTW			
	*OPT*	*KBS*	*Best.Sol*	*Aver.Sol*	*Gap*	*Time*	*Best.Sol*	*Aver.Sol*	*Gap*	*Time*
n60w160.004	401	11,645	401	401	0.0	19	11,778	11,778	1.1	19
n60w180.002	399	12,015	399	399	0.0	17	12,224	12,224	1.7	21
n60w180.003	445	12,214	445	445	0.0	18	12,214	12,679	0.0	21
n60w180.004	456	11,101	456	456	0.0	19	11,245	11,245	1.3	18
n60w200.002	414	11,748	414	414	0.0	20	11,866	11,866	1.0	19
n60w200.003	455	10,697	460	460	1.1	19	10,697	10,697	0.0	18
n60w200.004	431	11,441	431	431	0.0	16	11,441	11,441	0.0	17
n80w120.002	577	18,181	577	577	0.0	17	18,383	18,383	1.1	21
n80w120.003	540	17,878	548	548	1.5	19	17,937	17,937	0.3	21
n80w120.004	501	17,318	501	501	0.0	18	17,578	17,578	1.5	18
n80w140.002	472	17,815	472	472	0.0	19	17,815	17,815	0.0	21
n80w140.003	580	17,315	580	580	0.0	20	17,358	17,358	0.2	21
n80w140.004	424	18,936	424	424	0.0	19	18,936	18,936	0.0	18
n80w160.002	553	17,091	553	553	0.0	27	17,200	17,200	0.6	18
n80w160.003	521	16,606	521	521	0.0	21	16,521	16,521	−0.5	20
n80w160.004	509	17,804	509	509	0.0	20	17,927	17,927	0.7	18
n80w180.002	479	17,339	479	479	0.0	21	17,339	17,339	0.0	19
n80w180.003	530	17,271	530	530	0.0	20	17,160	17,160	−0.6	20
n80w180.004	479	16,729	479	479	0.0	22	16,849	16,849	0.7	21
n100w120.002	540	29,882	556	556	3.0	38	29,818	29,818	0.0	45
n100w120.003	617	25,275	646	646	4.7	37	24,473	24,473	0.0	42
n100w120.004	663	30,102	663	663	0.0	39	31,554	31,554	0.0	41
n100w140.002	622	30,192	632	632	1.6	38	30,087	30,087	0.0	45
n100w140.003	481	28,309	481	481	0.0	39	28,791	28,791	0.0	47
n100w140.004	533	27,448	533	533	0.0	40	27,990	27,990	0.0	45
aver					0.64	13.6			−0.44	14.7

**Table 8:** Comparison between our results with the best-found values for TSPTW and TRPTW instances proposed by Gendreau et al. (1998), and Ohlmann & Thomas (2007).

Instances	TSPTW	TRPTW		MFEA+RNVS					
				TSP			TRP		
	*OPT*	BKS		*Best.Sol*	*Aver.Sol*	*Time*	*Best.Sol*	*Aver.Sol*	*Time*
		Ban (2021)	Heilporna, Cordeaua & Laporte (2010)						
n20w120.001	274	2,175	2,535	274	274	2	2,175	2,175	2
n20w140.001	176	1,846	1,908	176	176	2	1,826	1,826	2
n20w160.001	241	2,146	2,150	241	241	2	2,148	2,148	2
n20w180.001	253	2,477	2,037	253	253	2	2,477	2,477	2
n20w200.001	233	1,975	2,294	233	233	2	1,975	,1975	2
n40w120.001	434	6,800	7,496	434	434	9	6,800	6,800	9
n40w140.001	328	6,290	7,203	328	328	10	6,290	6,290	10
n40w160.001	349	6,143	6,657	349	349	11	6,143	6,143	12
n40w180.001	345	6,952	6,578	345	345	12	6,897	6,897	11
n40w200.001	345	6,169	6,408	345	345	10	6,113	6,113	13
n60w120.001	392	11,120	9,303	392	392	25	11,288	11,288	28
n60w140.001	426	10,814	9,131	426	426	26	10,981	10,981	27
n60w160.001	589	11,574	11,422	589	589	27	11,546	11,546	28
n60w180.001	436	11,363	9,689	436	436	24	11,646	11,646	25
n60w200.001	423	10,128	10,315	423	423	25	9,939	9,939	27
n80w120.001	509	11,122	11,156	512	509	41	16,693	16,693	45
n80w140.001	530	14,131	14,131	530	530	42	14,131	14,131	47
n80w180.001	605	11,222	11,222	605	605	41	11,222	11,222	42

**Table 9:** The average results for TSPTW, TRPTW instances.

Instances	TSPTW			TRPTW
	$\overline {gap}$	*Time*	$\overline {gap}$	*Time*
TSPTW	0.49	26.24	−0.11	26.5
TSPTW	0.64	13.6	−0.44	14.7
aver	0.56	19.9	−0.28	20.6

### Evaluating the efficiency of selection

In this experiment, a new selection operator for the MFEA+RVNS algorithm effectively balances knowledge transfer and diversity. Due to being too expensive in computation, we choose some instances to evaluate the efficiency of this operator. In Table 2, the column MFEA-NR results from the MFEA+RVNS with the selection-based scalar-fitness only, while column MFEA+RVNS is the results of the MFEA+RVNS with both scalar-fitness and diversity. The $diff[\% ]$ column is the difference between the MFEA+RVNS and MFEA-NR in percentage.

In Table 2, the MFEA+RVNS outperforms the MFEA-NR in all cases. The selection operation that considers both scalar-fitness and diversity to pick parents is more effective than the one with scalar-fitness only. The fitness-based criterion promotes “survival of the fittest”, which is good for transferring elite genes between tasks, and good individuals are kept in each task, implying an accumulation of good genes. However, population diversity is important since it becomes a bottleneck. Our selection ensures that selection recognizes both diversity contribution and fitness in choosing the best individuals for reproduction.

### Evaluating the balance between exploration and exploitation

Generally speaking, algorithms get stuck into local optimum because there is a lack of balance between exploration and exploitation. Exploration helps the search to explore extension spaces on a global scale, while exploitation helps the search to focus on local space by exploiting the information that a current solution is reached in this space. In this experiment, the balance between exploration and exploitation is considered. We run the MFEA with or without the RVNS on the same selected instances. In Table 3, the MFEA-NLS column is the MFEA without the RVNS, while the MFEA+RVNS column is the MFEA with the RVNS. The $diff[\% ]$ column is the difference between the MFEA+RVNS and MFEA-NLS.

**Figure 4:**
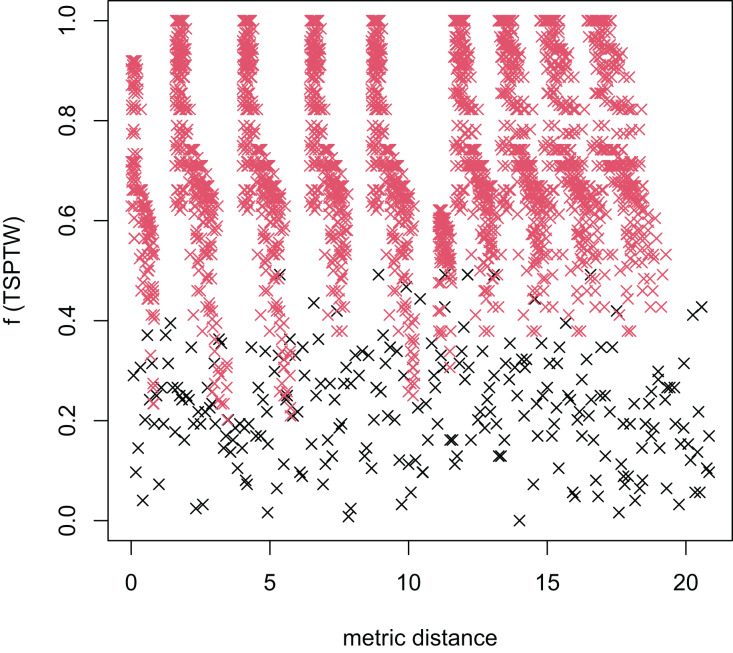
The average distance to the other local optima in n20w100.002 instance for the TSPTW.

**Figure 5:**
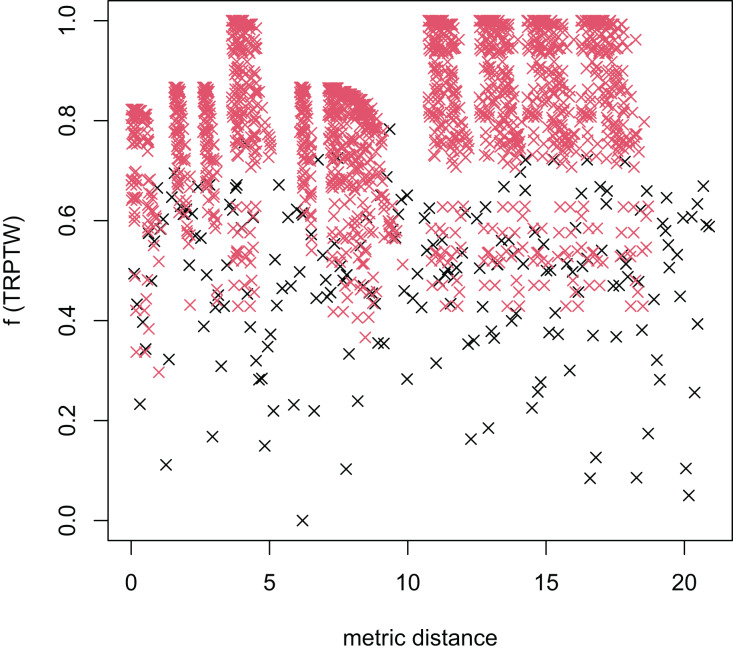
The average distance to the other local optima in n20w100.002 instance for the TRPTW.

**Figure 6:**
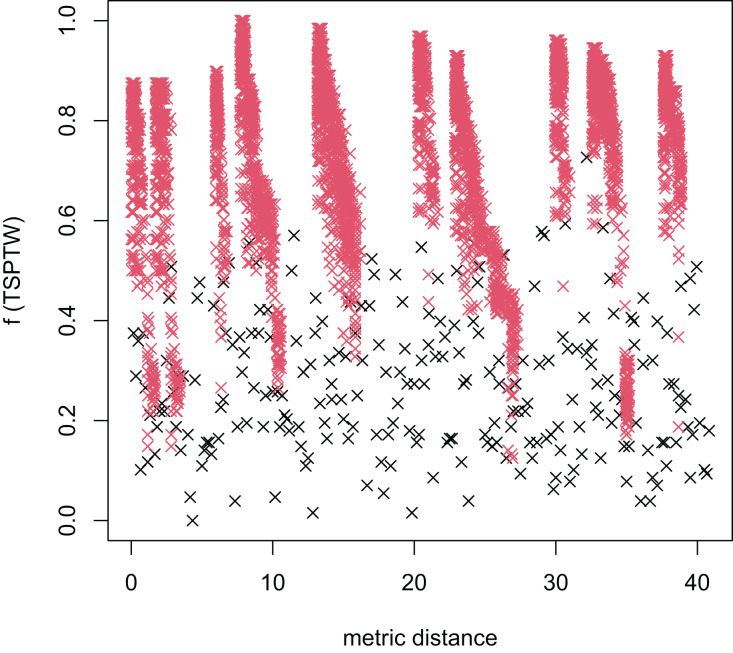
The average distance to the other local optima in n40w100.002 instance for the TSPTW.

**Figure 7:**
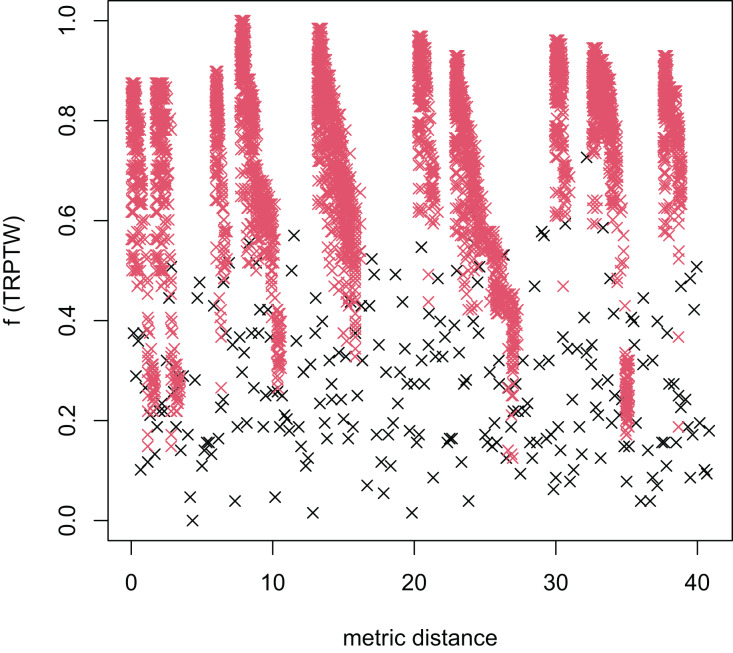
The average distance to the other local optima in n40w100.002 instance for the TRPTW.

In Table 3, the MFEA+RVNS obtains much better solutions than the MFEA-NLS in all cases. It indicates that the combination between the MFEA and RNVS has a good balance between exploration and exploitation. To study the ability to balance exploration and exploitation of the search space, we implement an experimental study on the distribution of locally optimal solutions. We choose two instances (n20w100.002 and n40w100.002) to perform one execution of our algorithm and record the distinct local optima encountered in some generations. We then plot the normalized tour’s cost *vs* its average metric distance to all other local minima (the distance metric and its average is defined in “Selection operator”). The results are illustrated in Figs. 4–7. The black “x” points indicate the result of the MFEA, while the red “x” points show the results of the RNVS. The normalized cost can be used as follows:

(4) $$\overline {{f_j}} = \displaystyle{{({f_j} - f_j^{min})} \over {(f_j^{max} - f_j^{min})}},$$

where $j = 1,2$ is the $j$-task and $f_j^{min},f_j^{max}$ are the minimum and maximum cost values for all runs, respectively.

Figures 4–7 show that the black “x” points are spread quite widely, which implies that the proposed algorithm has the power to search over a wide region of the solution space. It is the capacity for exploration. On the other hand, the red points describe the search tends to exploit the good solution space explored by the MFEA. It is the capacity for exploitation. As a result, the algorithm maintains the right balance between exploration and exploitation.

### Comparisons with the TSPTW and TRPTW algorithms

In Table 9, the average difference with the optimal solution for the TSPTW is 0.56%, even for instances with up to 200 vertices. It shows that our solutions are near-optimal for the TSPTW. In addition, the proposed algorithm reaches the optimal solutions for the instances with up to 80 vertices for the TSPTW. In Table 8, for the TRPTW, our MFEA+RVNS is better than the previous algorithms such as Ban & Nghia (2017), Ban (2021) and Heilporna, Cordeaua & Laporte (2010) in the literature when the average *gap* is −0.28% (note that: Ban et al.’s and Heilporna et al.’s (2010) algorithms is developed to solve the TRPTW only). The obtained results are impressive since it can be observed that the proposed algorithm finds not only near-optimal solutions but also the new best-known ones for two problems simultaneously. It also indicates the efficiency of positive transferrable knowledge control techniques between optimization tasks in improving the solution quality.

It is impossible to expect that the MFEA+RVNS always outperform in comparison with the state-of-the-art metaheuristic algorithms for the TSPTW and TRPTW in all cases because their algorithms are designed to solve each problem independently. Table 10 shows that the efficient algorithms for the TSPTW may not be effective for the TRPTW and vice versa. On average, the optimal solution for the TSPTW with the TRPTW objective cost is about 9.7% worse than the optimal one for the TRPTW. Similarly, the known best solution for the TRPTW using the TSPTW objective function is 20.6% worse than the optimal solution for the TSPTW. We conclude that if the proposed MFEA simultaneously reaches good solutions that are close to the optimal solutions for both problems and even better than the state-of-the-art algorithms in many cases, we can say that the proposed MFEA+RVNS for multitasking is beneficial.

**Table 10:** The difference between the optimal TSPTW using TRPTW objective function and *vice versa*.

Instances	TRPTW			TSPTW		
	TRPTW (OPT_TSPTW)	KBS	diff[%]	TSPTW (BKS_TRPTW)	KBS	diff[%]
n20w120.002	2,592	2,193	15.4	256	218	14.8
n20w140.002	2,519	2,330	7.5	311	272	12.5
n20w160.002	2,043	1,830	10.4	249	201	19.3
n40w120.002	6,718	6,265	6.7	552	446	19.2
n40w140.002	5,865	5,746	2.0	449	383	14.7
n40w160.002	7,519	6,351	15.5	456	338	25.9
n60w120.002	13,896	1,2517	9.9	581	444	23.6
n60w140.002	12,898	11,795	8.6	616	464	24.7
n60w160.002	14,091	12,489	11.4	616	428	30.5
aver			9.7			20.6

### Comparison with the previous MFEA algorithms

We adopt the proposed algorithm in the experiment to solve the TSP and TRP problems. Otherwise, we also use three algorithms (Ban & Pham, 2022; Osaba et al., 2020; Yuan et al., 2016) to solve the TSPTW and TRPTW.

Tables 11–13 compare our results to those of three algorithms (Ban & Pham, 2022; Osaba et al., 2020; Yuan et al., 2016) for some instances in both the TSP and TRP problems. The results show that the proposed algorithm obtains better solutions than the others in all cases. The difference between our average result and the optimal value is below 2.59%. It shows that our solution is the very near-optimal one. In addition, our algorithm reaches the optimal solution for the instance with 76 vertices. Obviously, the proposed algorithm applies well in the case of the TSP and TRP.

**Table 11:** Comparison between our results with the others for TSP and TRP (Yuan et al., 2016).

Instances	OPT		YA (Yuan et al., 2016)		OA (Tsitsiklis, 1992)		MFEA+RNVS							
	TSP	TRP	TSP	TRP	TSP	TRP	TSP				TRP			
			*best.sol*	*best.sol*	*best.sol*	*best.sol*	*best.sol*	*aver.sol*	*Gap*	*Time*	*best.sol*	*aver.sol*	*Gap*	*Time*
eil51	426*	10,178*	446	10,834	450	10,834	426	426	0.00	23	10,178	10,178	0.00	22
berlin52	7,542*	143,721*	7,922	152,886	8,276	152,886	7,542	7,542	0.00	22	143,721	143,721	0.00	21
st70	675*	20,557*	713	22,283	772	22,799	680	680	0.01	41	22,283	22,283	8.40	39
eil76	538*	17,976*	560	18,777	589	18,008	559	559	0.04	43	18,008	18,008	0.18	40
pr76	108,159*	3,455,242*	113,017	3,493,048	117,287	3,493,048	108,159	108,159	0.00	47	3,455,242	3,455,242	0.00	45
pr107	44,303*	2,026,626*	45,737	2,135,492	46,338	2,135,492	45,187	45,187	0.02	71	2,052,224	2,052,224	1.26	72
rat99	1,211*	58,288*	1,316	60,134	1,369	60,134	1,280	1,280	0.06	66	58,971	58,971	1.17	65
kroA100	21,282*	983,128*	22,233	1,043,868	22,233	1,043,868	21,878	21,878	0.03	63	1,009,986	1,009,986	2.73	63
kroB100	22,141*	986,008*	23,144	1,118,869	24,337	1,118,869	23,039	23,039	0.04	64	1,003,107	1,003,107	1.73	63
kroC100	20,749*	961,324*	22,395	1,026,908	23,251	1,026,908	21,541	21,541	0.04	68	1,007,154	1,007,154	4.77	66
kroD100	21,294*	976,965*	22,467	1,069,309	23,833	1,069,309	22,430	22,430	0.05	70	1,019,821	1,019,821	4.39	72
kroE100	22,068*	971,266*	22,960	1,056,228	23,622	1,056,228	22,964	22,964	0.04	60	1,034,760	1,034,760	6.54	64
rd100	7,910*	340,047*	8,381	3,80,310	8,778	365,805	8,333	8,333	0.05	63	354,762	354,762	4.33	64
eil101	629*	27,519*	681	28,398	695	28,398	662	662	0.05	62	27,741	27,741	0.81	61
aver									0.03				2.59	

**Note:**

* Indicates the optimal values.

**Table 12:** Comparison between our results with the others for TSP and TRP in TRP-50-x.

Instances	OPT		YA		OA		MFEA+RNVS	
	TSP	TRP	TSP	TRP	TSP	TRP	TSP	TRP
	best.sol	best.sol	best.sol	best.sol	best.sol	best.sol	best.sol	best.sol
TRP-50-1	602	12,198	641	13,253	634	13,281	610	12,330
TRP-50-2	549	11,621	583	12,958	560	12,543	560	11,710
TRP-50-3	584	12,139	596	13,482	596	13,127	592	12,312
TRP-50-4	603	13,071	666	14,131	613	15,477	610	13,575
TRP-50-5	557	12,126	579	13,377	578	14,449	557	12,657
TRP-50-6	577	12,684	602	13,807	600	13,601	588	13,070
TRP-50-7	534	11,176	563	11,984	555	12,825	547	11,793
TRP-50-8	569	12,910	629	14,043	609	13,198	572	13,198
TRP-50-9	575	13,149	631	14,687	597	13,459	576	13,459
TRP-50-10	583	12,892	604	14,104	602	13,638	590	13,267
TRP-50-11	578	12,103	607	13,878	585	12,124	585	12,124
TRP-50-12	500	10,633	521	11,985	508	11,777	604	11,305
TRP-50-13	579	12,115	615	13,885	601	13,689	587	12,559
TRP-50-14	563	13,117	612	14,276	606	14,049	571	13,431
TRP-50-15	526	11,986	526	12,546	526	12,429	526	12,429
TRP-50-16	551	12,138	577	13,211	564	12,635	551	12,417
TRP-50-17	550	12,176	601	13,622	585	13,342	564	12,475
TRP-50-18	603	13,357	629	14,750	625	14,108	603	13,683
TRP-50-19	529	11,430	595	12,609	594	12,899	539	11,659
TRP-50-20	539	11,935	585	13,603	575	12,458	539	12,107

**Table 13:** Comparison between our results with the others for TSP and TRP in TRP-100-x.

Instances	OPT		YA		OA		MFEA+RNVS	
	TSP	TRP	TSP	TRP	TSP	TRP	TSP	TRP
	best.sol	best.sol	best.sol	best.sol	best.sol	best.sol	best.sol	best.sol
TRP-100-1	762	32,779	830	36,012	806	36,869	791	35,785
TRP-100-2	771	33,435	800	39,019	817	37,297	782	35,546
TRP-100-3	746	32,390	865	38,998	849	34,324	767	34,324
TRP-100-4	776	34,733	929	41,705	897	38,733	810	37,348
TRP-100-5	749	32,598	793	40,063	899	37,191	774	34,957
TRP-100-6	807	34,159	905	40,249	886	40,588	854	36,689
TRP-100-7	767	33,375	780	38,794	849	39,430	780	35,330
TRP-100-8	744	31,780	824	38,155	845	35,581	763	34,342
TRP-100-9	786	34,167	863	39,189	858	41,103	809	35,990
TRP-100-10	751	31,605	878	36,191	831	37,958	788	33,737
TRP-100-11	776	34,188	831	39,750	876	41,153	814	36,988
TRP-100-12	797	32,146	855	39,422	855	40,081	823	34,103
TRP-100-13	753	32,604	772	37,004	772	40,172	771	35,011
TRP-100-14	770	32,433	810	40,432	810	36,134	800	34,576
TRP-100-15	776	32,574	953	38,369	878	38,450	810	35,653
TRP-100-16	775	33,566	838	40,759	835	38,549	808	36,188
TRP-100-17	805	34,198	939	39,582	881	42,155	838	36,969
TRP-100-18	785	31,929	876	38,906	836	37,856	814	34,154
TRP-100-19	780	33,463	899	39,865	881	40,379	797	35,669
TRP-100-20	775	33,632	816	41,133	905	40,619	808	35,532

Statistical tests are used to check whether the difference between the proposed algorithm and the remaining ones is significant or not. A non-parametric test (Friedman and Quad test) is carried out in the group of the algorithms to check if a significant difference between them is found. The output of the Friedman test in Table 14 illustrates the rankings achieved by the Friedman and Quade tests. The results in this table strongly indicate considerable differences between the algorithms. Because the YA and OA have the largest ranking, the MFEA+RVNS is selected as the control algorithms. After that, we compare the control algorithms with YA and OA by statistical methods. Table 15 shows the possible hypotheses of comparison between the control algorithm and other algorithms. The statistical result shows that the MFEA+RVNS outperforms YA and OA with a level of significance $\alpha = 0.05$.

**Table 14:** Average rankings of the algorithms.

Algorithm	TSP		TSP	
	Friedman	Quade	Friedman	Quade
YA	2.73	2.70	2.59	2.52
OA	2.15	2.18	2.33	2.43
MFEA+RNVS	1.11	1.11	1.06	1.04

**Table 15:** The *z*-values and *p*-values of the Friedman procedures (MFEA+RNVS is the control algorithm) in both the TSP and TRP.

*i*	Algorithm	TSP					TRP				
		*z*	*p*	Holm	Holland	Rom	*z*	*p*	Holm	Holland	Rom
1	YA	7.26	3.66E−13	0.025	0.025	0.025	6.87	6.15E−12	0.025	0.025	0.025
2	OA	4.63	3.48E−06	0.05	0.05	0.05	5.70	1.18E−08	0.05	0.05	0.05

**Figure 8:**
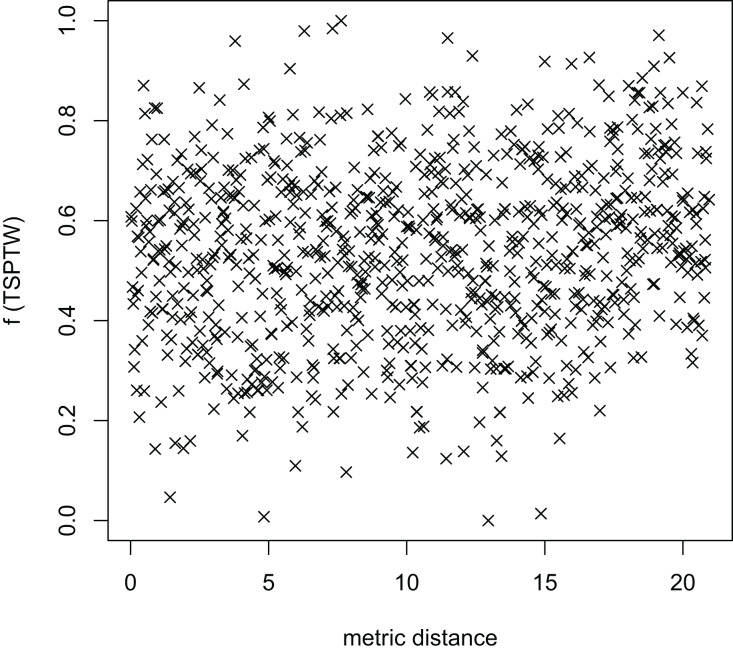
The average distance to the other local optima in n20w100.002 instance for the TSP (YA algorithm).

**Figure 9:**
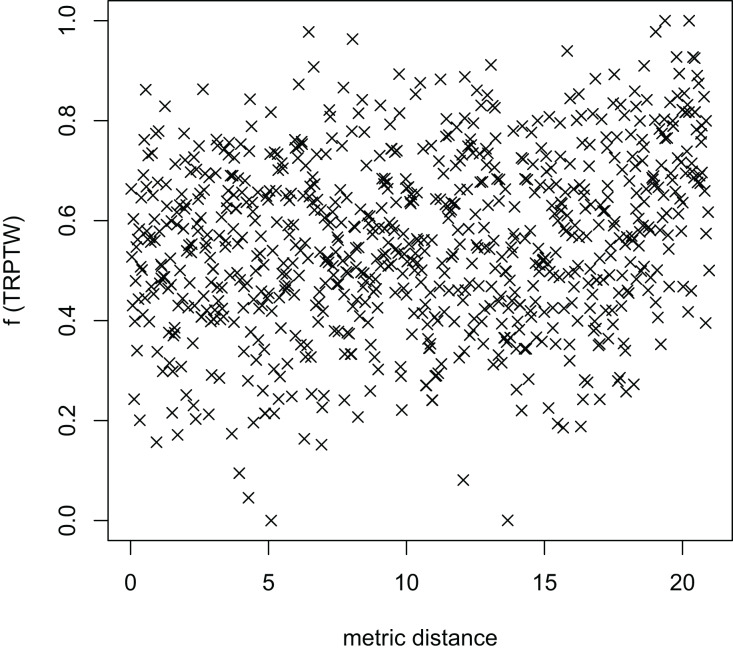
The average distance to the other local optima in n20w100.002 instance for the TRP (YA algorithm).

**Figure 10:**
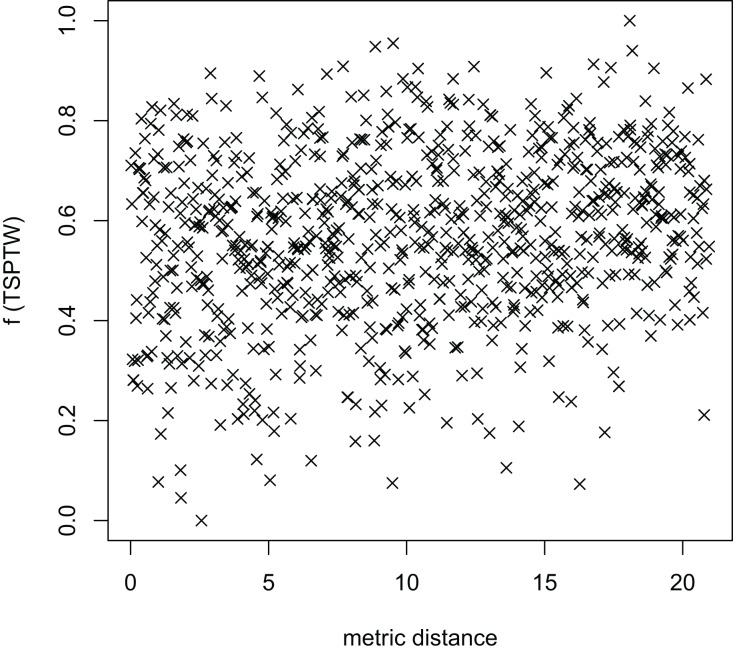
The average distance to the other local optima in n20w100.002 instance for the TSP (OA algorithm).

**Figure 11:**
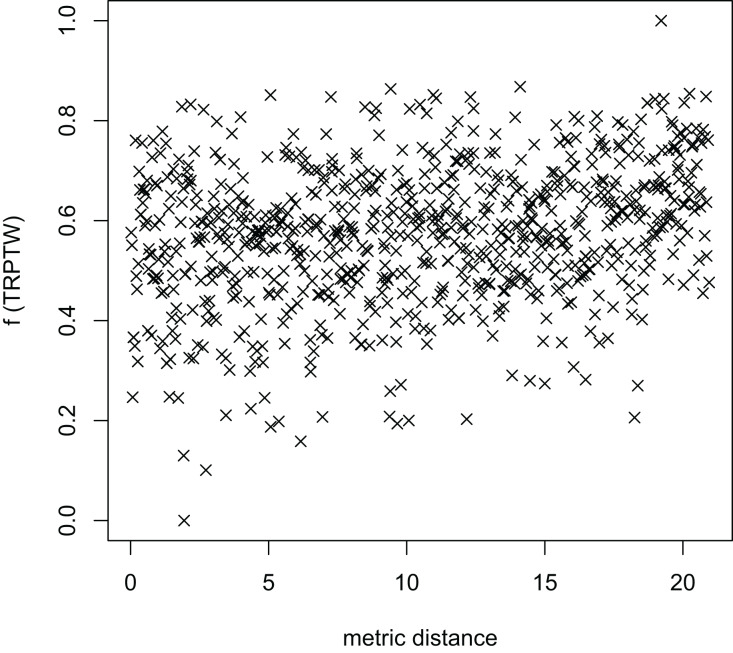
The average distance to the other local optima in n20w100.002 instance for the TRP (OA algorithm).

**Figure 12:**
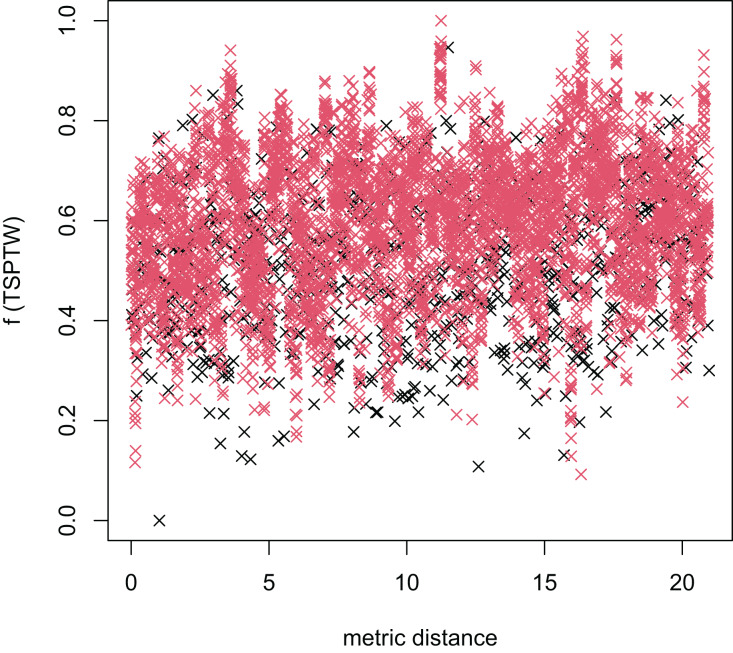
The average distance to the other local optima in n20w100.002 instance for the TSP (the proposed algorithm).

**Figure 13:**
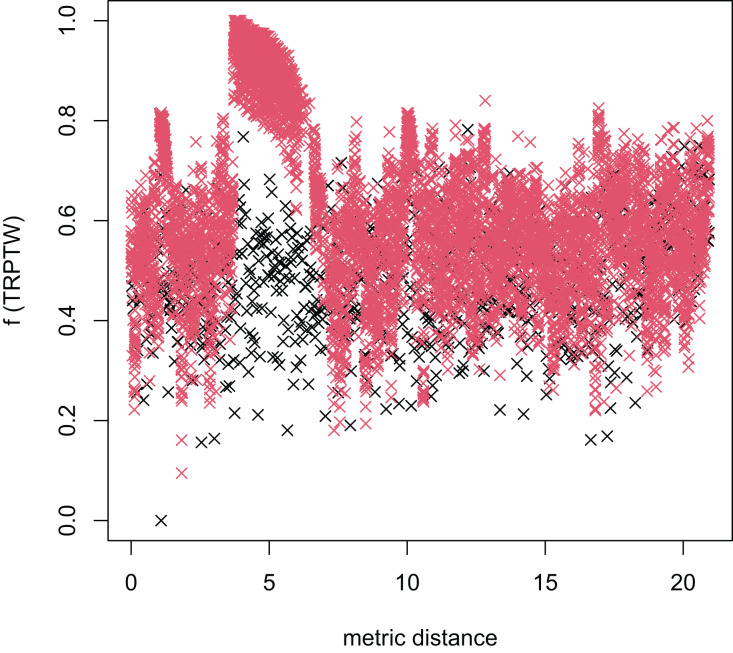
The average distance to the other local optima in n20w100.002 instance for the TRP (the proposed algorithm).

Figures 8–13 describe the normalized tour’s cost *vs* its average metric distance to all other local minima of two algorithms (YA and OA). The black and red points are still the results of the MFEA and RVNS, respectively. Their algorithm explores widely in the solution space, implying a good capacity for exploration. However, exploitation capacity is not good enough. Figures 12 and 13 show that the RVNS exploits good solution spaces explored by the MFEA much better. It is understandable when the proposed algorithm reaches better results than theirs.

Moreover, Table 16 compares our results to those of three algorithms (Ban & Pham, 2022; Osaba et al., 2020; Yuan et al., 2016) in both the TSPTW and TRPTW problems. The results show that three algorithms (Ban & Pham, 2022; Osaba et al., 2020; Yuan et al., 2016) cannot find feasible solutions while the proposed algorithm reaches feasible ones in all cases. It is understandable because these algorithms drive the search for solution spaces that maybe not contain feasible solutions. Otherwise, the proposed algorithm guides the search process to feasible solution spaces. It is an important contribution because finding a feasible solution for the TSPTW, and TRPTW is even NP-hard (Ban, 2021).

**Table 16:** Comparison between our results with the others (Ban & Pham, 2022; Osaba et al., 2020; Yuan et al., 2016) for TSPTW and TRPTW.

Instances	YA (Xu et al., 2021)		OA (Tsitsiklis, 1992)		BP (Ban & Pham, 2022)		MFEA+RNVS	
	TSPTW	TRPTW	TSPTW	TRPTW	TSPTW	TRPTW	TSPTW	TRPTW
n40w40.002	–	–	–	–	–	–	461	7,104
n40w60.002	–	–	–	–	–	–	470	7,247
n40w80.002	–	–	–	–	–	–	431	7,123
n40w100.002	–	–	–	–	–	–	364	6,693
n60w20.002	–	–	–	–	–	–	605	13,996
n60w120.002	–	–	–	–	–	–	427	12,525
n60w140.002	–	–	–	–	–	–	464	11,810
n60w160.002	–	–	–	–	–	–	423	12,719
n80w120.002	–	–	–	–	–	–	577	18,383
n80w140.002	–	–	–	–	–	–	472	18,208
n80w160.002	–	–	–	–	–	–	553	17,200
n100w120.002	–	–	–	–	–	–	556	29,818
n100w140.002	–	–	–	–	–	–	632	30,087
n100w120.003	–	–	–	–	–	–	646	24,473
n100w140.003	–	–	–	–	–	–	481	28,791
n100w140.004	–	–	–	–	–	–	533	27,990

### Convergence trend

**Figure 14:**
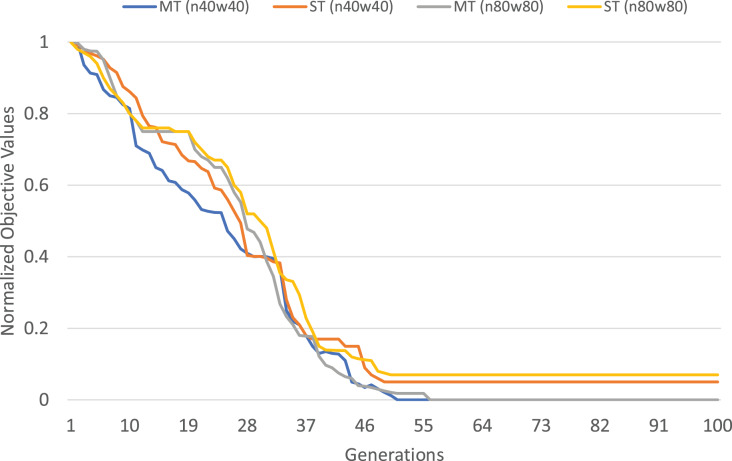
Comparing convergence trends of $ f_1$ in multi-tasking and single-tasking for the TSPTW in n40w40 and n80w80 instances.

**Figure 15:**
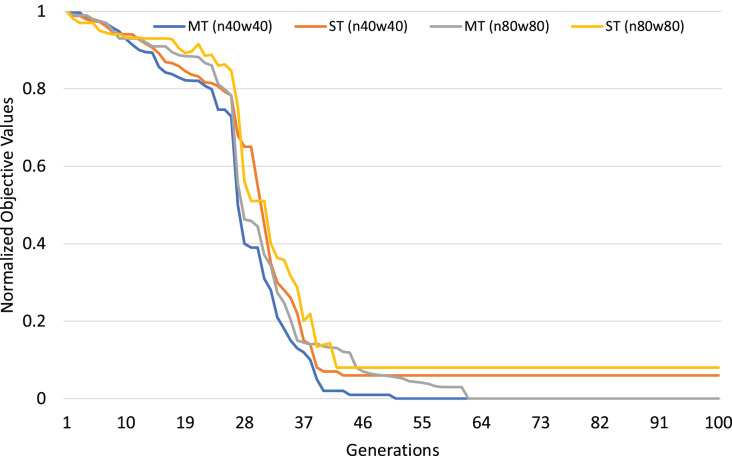
Comparing convergence trends of $f_2$ in multi-tasking and single-tasking for the TRPTW in n40w40 and n80w80 instances.

The normalized objective cost (see formulation 4) can be used to analyze the convergence trends of our MFEA+RVNS algorithm. The convergence trend of the two strategies is described in Figs. 4 and 5 for n40w40 and n80w80 instances. The x-axis describes the number of generations, while the y-axis illustrates the normalized objective value. The less and less the normalized objective value is, the better and better the algorithm is. In Figs. 14 and 15, Single-tasking (ST) converges better than multitasking (MT) in the whole evolutionary process while avoiding premature convergence to sub-optimal solutions by exchanging knowledge among tasks. That means, in general, multitasking converges to a better objective value.

When multitasking is run with the same number of generations as single-tasking, on average, it only consumes $\displaystyle{1 \over K}$ computational effort for each task (*K* is the number of tasks). Therefore, we consider the worst-case situation when the number of generations for multitasking is *K* times the one for single-tasking. If multitasking obtains better solutions than single-tasking in this case, we can say that multitasking saves computational efforts. The experimental results are described in Table 17. In Table 17, the first row shows the average gap of single-task for the TSPTW and TRPTW, while the second shows the average gap of multitasking with 100 generations. The result shows that multitasking consumes only $\displaystyle{1 / 2}$ computational efforts to obtain better solutions than single-tasking.

**Table 17:** Comparison computational effort between single-tasking and multitasking.

Type	TSPTW	TRPTW
	*gap*	*gap*
Single-tasking (100 generations)	0.59	0.08
Multi-tasking (100 generations)	0.56	−0.28

In short, the efficiency of multitasking is better in comparison with single-tasking because of the process of transferring knowledge during multitasking. It is an impressive advantage of the evolutionary multitasking paradigm.

## Conculsions and future work

In this article, our contribution is threefold. Firstly, we propose a new selection operator that balances skill-factor and population diversity. The skill-factor effectively transfers elite genes between tasks, while diversity in the population is important when it meets a bottleneck against the information transfer. Secondly, multiple crossover schemes are applied in the proposed MFEA+RVNS. They help the algorithm have good enough diversity. In addition, two types of crossover (intra- and inter-) are used. It opens up the chance for knowledge transfer through crossover-based exchange between tasks. Lastly, the combination between the MFEA and the RVNS is to have good transferrable knowledge between tasks from the MFEA and the ability to exploit good solution spaces from the RVNS. However, focusing only on reducing cost function maybe lead the search to infeasible solution spaces like the algorithm (Ban & Pham, 2022). Therefore, the repair technique is incorporated into the proposed algorithm to balance finding feasible solution spaces and reducing cost function.

Extensive experiments on the benchmark dataset indicate that the proposed algorithm simultaneously obtains good solutions for both problems. In addition, it obtains better solutions than the other MFEA algorithms in many cases. More interestingly, it finds the new best-known solutions compared to the state-of-the-art metaheuristics only for the TRPTW in many cases.

In future work, we will study how to apply multiple population ideas for multitasking. Many researchers is interested in the approach (Chen et al., 2020; Li, Zhang & Gao, 2018; Wei & Zhong, 2020; Wei et al., 2022; Xu et al., 2021). The approach’s advantages include: (1) each population evolves with different genetic operators, and each individual can be represented differently; (2) individuals migrate between populations. The approach maintains diversity and improves convergence trends.

## Additional Information and Declarations

### Data Availability

The following information was supplied regarding data availability: The data and code are available at Zenodo: Ha-Bang Ha, & Dang-Hai Pham. (2022). Dataset and code for the variants of the traveling salesman problem with time windows using multifactorial evolutionary algorithm [Data set]. In PeerJ Computer Science. Zenodo. https://doi.org/10.5281/zenodo.7331323.
